# Association between Dietary Nitrate, Nitrite Intake, and Site-Specific Cancer Risk: A Systematic Review and Meta-Analysis

**DOI:** 10.3390/nu14030666

**Published:** 2022-02-04

**Authors:** Kassim Said Abasse, Eno E. Essien, Muhammad Abbas, Xiaojin Yu, Weihua Xie, Jinfang Sun, Laboni Akter, Andre Cote

**Affiliations:** 1 Faculté des Sciences de L’Administration FSA, Université Laval, Québec, QC G1V 0A6, Canada ; Kassim.said-abasse@fsa.ulaval.ca; 2 Department of Epidemiology and Biostatistics, School of Public Health, Southeast University, Nanjing 210009, China; essienenoernest@yahoo.com (E.E.E.); xie.weihua@foxmail.com (W.X.); sjf_1128@126.com (J.S.); 3 Riphah Institute of Pharmaceutical Sciences, Riphah International University, Islamabad 44000, Pakistan; 4 Institute of Epidemiology, Disease Control & Research (IEDCR), Dhaka 1212, Bangladesh; labonia608@gmail.com

**Keywords:** nitrate, nitrites, dietary intake, cancer, humans, systematic review

## Abstract

Background: People consume nitrates, nitrites, nitrosamines, and NOCs compounds primarily through processed food. Many studies have yielded inconclusive results regarding the association between cancer and dietary intakes of nitrates and nitrites. This study aimed to quantify these associations across the reported literature thus far. Methods: We performed a systematic review following PRISMA and MOOSE guidelines. A literature search was performed using Web of Science, Embase, PubMed, the Cochrane library, and google scholar up to January 2020. STATA version 12.0 was used to conduct meta-regression and a two-stage meta-analysis. Results: A total of 41 articles with 13 different cancer sites were used for analysis. Of these 13 cancer types/sites, meta-regression analysis showed that bladder and stomach cancer risk was greater, and that pancreatic cancer risk was lower with increasing nitrite intakes. Kidney and bladder cancer risk were both lower with increasing nitrate intakes. When comparing highest to lowest (reference) categories of intake, meta-analysis of studies showed that high nitrate intake was associated with an increased risk of thyroid cancer (OR = 1.40, 95% CI: 1.02, 1.77). When pooling all intake categories and comparing against the lowest (reference) category, higher nitrite intake was associated with an increased risk of glioma (OR = 1.12, 95% CI: 1.03, 1.22). No other associations between cancer risk and dietary intakes of nitrates or nitrites were observed. Conclusion: This study showed varied associations between site-specific cancer risks and dietary intakes of nitrate and nitrite. Glioma, bladder, and stomach cancer risks were higher and pancreatic cancer risk was lower with higher nitrite intakes, and thyroid cancer risk was higher and kidney cancer risk lower with higher nitrate intakes. These data suggest type- and site-specific effects of cancer risk, including protective effects, from dietary intakes of nitrate and nitrite.

## 1. Introduction

Cancer is a leading cause of death worldwide, accounting for nearly 10.0 million (approximately one in six) deaths, in 2020 [1]. The global cancer burden is expected to be 28.4 million cases in 2040, a 47% rise from 2020 [1]. With this growing global burden, more evidence-based practice is needed in the identification and management of risk factors for cancer development. Although the causes of cancer are not completely understood, numerous factors are known to increase risk, including non-modifiable factors (e.g., gender, age, genetic factors) and modifiable factors (e.g., dietary, lifestyle) [2]. For instance, one-third of deaths from cancer are due to behavioral and dietary risks [3]. 

Over the past few years, new evidence has led a paradigm change in our understanding of the role of both dietary nitrate and nitrite on human health, particularly in relation to cancer risk [4,5,6,7]. Historically, a high intake of nitrate and nitrite were considered harmful food additives and listed as probable human carcinogens under conditions where endogenous nitrosation could take place and lead to formation of *N*-nitroso compounds (NOC) [2,4,8]. Nowadays, nitrate, nitrite and nitrosamine occur naturally in fruits and vegetables, which are regarded as an important part of a healthy diet due to the powerful evidence of beneficial health effects against cancer [9,10]. Numerous studies concluded that fruits and vegetables contribute over 80% of the daily dietary intake of nitrate [11,12,13] and nitrite [14,15,16,17], which represent the primary sources of exposure to nitrate and nitrite by humans. 

Nitrates and nitrites are also widely used in food processing, such as in processed meats (e.g., sausages, hot dogs, luncheon meats, ham, and bacon) where they are used to reduce microbial spoilage and preserve meat products [18,19]. High consumption of both processed and fresh meat is linked to increased cancer risk (e.g., gastric [20], pancreatic [21], bladder [5], and colorectal [19]). It is the presence of nitrite, amides, and amines [8,22,23,24] in processed meats and heme iron in fresh meat [25,26,27,28,29] that is considered to be responsible for these risk effects. Moreover, there are other biological and dietary factors that may contribute to the effects that nitrates, nitrites, and their related compounds have on the body. For example, certain strains of oral bacteria have been identified that reduce nitrate found in food to nitrite [30]. This ”endogenous nitrosation” is known to occur mostly in the digestive system’s organs, especially the stomach, rectum, colon, and urinary bladder [31,32], but it can take place in any part of the body. Some of the NOC’s forms have been identified in human urine [33].

Despite the carcinogenic potential of NOCs, some epidemiologic studies found no association between dietary nitrate, nitrite, and NOC intake and cancer in humans [34,35,36]. Case control studies in Iowa [35] and Spain [36] found no association between long-term, average nitrate levels in public water supplies and bladder cancer. This might be due to the fact that nitrate in drinking water and their related compounds are in low concentrations [35,36,37]. Overall, epidemiologic studies that have examined associations between nitrate, nitrite, and NOC compounds and various types of cancer in humans have returned mixed and, in some cases, complex results. Some studies show positive associations [5,6,20,38,39], many show no association [35,36,37], and others show inverse associations, which may be due to confounding factors [31,37,40,41,42,43,44]. In this study, this systematic review aims to evaluate the association and clarify the relationship between dietary consumption of nitrate and nitrite and selected, site-specific cancer risks in humans. 

## 2. Methods 

Meta-analyses are typically used to estimate the overall/mean of an outcome of interest. However, inference about between-study variability, which is typically modeled using a between-study variance parameter, is usually an additional aim [45]. Meta-regression is a sensitivity analysis. In primary studies, we use regression, or multiple regression, to assess the relationship between one or more covariates (moderators) and a dependent variable. The same approach can be used with meta-analysis, except that the covariates are at the level of the study rather than the level of the subject, and the dependent variable is the effect size in the studies rather than subject scores. We use the term meta-regression to refer to these procedures when used in a meta-analysis [46].

### 2.1. Search Methods for Identifications of Studies

We performed a systematic review following PRISMA and MOOSE guidelines. This study evaluated the association and clarified the relationship between dietary nitrate, nitrite, and selected, site-specific cancers. Two investigators independently searched literature on the Web of Science, Embase, PubMed, the Cochrane library, and google scholar up to January 2020. 

### 2.2. The Keywords and Search Terms Used 

A literature search was performed on all the databases by using the following keywords: (Nitrate OR nitrates NO_3_-N OR NO_3_- OR nitrite OR nitrites OR N-nitroso compounds OR NO_2_-N OR NO_2_- OR sodium nitrate OR NaNO3 OR sodium nitrite OR NaNO2 OR ammonium nitrate OR NH4NO3 OR nitrosamine OR Nitrite amine OR NH2NO2 OR DMNA OR NDMA OR NDBA OR NMEA OR NDEA OR NPIP OR NPYR OR NMOR OR NDPA) AND (Neoplasm OR Tumor OR Tumour OR Cancer OR Carcinoma OR Carcinogenesis OR Malignant OR Adenocarcinoma OR Non-Hodgkin lymphoma OR Glioma) AND (Dietary OR Diet OR food). The full search strategy is provided in the online Supplementary Table S1. The bibliographies of original studies, reviews and relevant conferences were manually searched. 

### 2.3. Inclusion and Exclusion Criteria 

Inclusion criteria: Only articles reporting associations or outcomes between dietary nitrate, nitrite, and cancer in humans were used (both qualitatively and quantitatively). Exclusion criteria: All articles with animal experiments, articles not published in English, articles with a short commentary, short notes, no data or no records, or incomplete results and letters were excluded. Search results were screened for inclusion and exclusion criteria by four authors.

### 2.4. Data Extraction and Quality Assessment 

Data collection and quality assessment processes were performed by four authors (Essien, Weihua, Kassim, and Abbas), and any disagreement was settled by group discussion. *Qualitative analysis:* the data were extracted using a self-developed data extraction form. For selected studies, data included the study characteristics: first author, year published and country, study design, exposure categories (nitrate and nitrite intake mg/day), reported Risk ratios (RR), odds ratios (OR), and hazard ratios (HR) with their 95% CIs, cancer sites, and adjustment. *Quantitative analysis:* the data included: Nitrate dosages and nitrite dosages from dietary intake; OR/RR/HR, with their 95% CI for each category of exposure.

The Newcastle-Ottawa Quality Assessment Scale (NOS) was used to assess the quality of the included studies. Scores ranged from 0 to 9; studies with a score ≥7 were seen as high-quality studies [47].

### 2.5. Statistical Analysis

Software: STATA version 12.0 (StataCorp LP, College Station, TX, USA) was used to conduct meta-regression and meta-analysis (of both binary and continuous outcome variables, (Effect/CI)). (Statistical calculations and figures were produced with this software).

Analysis of data: All nitrate and nitrite dosage, and their related compounds, OR, RR, and HR, with 95% CIs were extracted (both crude and adjusted OR, RR, and HR,). The RRs and HRs were assumed to be the accurate estimates of ORs. The median intake of nitrate and nitrite dosage was calculated from the range given, (formula = lowest dosage (lower limit) + highest dosage (upper limit) divided by two in each given quartile). When the median intake was given, the data was used directly. When the interval of a quartile of any category of nitrate or nitrite dosage was not provided, the width of the class interval of quartile before this quartile was used to calculate and estimate the interval, (when the shortest or longest category was open-ended, it was assumed that the open-ended interval length had the same length as the adjacent interval). Logarithm of the ORs (LogOR) was calculated. Standard error was also calculated, (formula = (log 95% CI upper limit value—log 95% CI lower limit value) divided by 3.92) using Microsoft Office Excel.

Conversion of nitrate units and nitrite dosage: mg/day was the standard unit for nitrite and nitrate dosage from dietary intake used for analysis because most of the articles used these units in their analyses. The recommended daily calorie intake in the US is around 2500 kcal for men and 2000 kcal for women [48]; for this analysis, 2500 calories (also referred to as kcal) was used for the standard conversion of data. (a) mg/1000 kcal was converted to mg/day (formula = the dosage in mg/1000 kcal multiplied by 2.5 (2500 kcal = mg/day)). (b) Mcg (nanogram) was converted to mg/day; (formula = the dosage in mcg or micro/1000 kcals or microgram/1000 kcals or µg/1000 kcal/day is divided by 0.001 to be converted into mg/1000 kcals and then multiplied by 2.5. (c) Mcg/day was converted to mg/day; (formula = the dosage in mcg/day is divided by 0.001).

Meta-regression: The parameters for meta-regression calculation were the dependent variable (y) = LogOR; covariates = median (the median intake of nitrate and nitrite dosage); and within-study variability = standard error was used for analysis. Meta-regression analysis was conducted to determine the associations between nitrate and nitrite exposure and cancer risk. A meta-regression coefficient was considered statistically significant at *p* ≤ 0.05. A sensitivity analysis was performed whereby extreme dosages were removed, including their ORs. Any cancer site that had less than three studies was not included in the meta-regression (these included ovarian cancer and uterus corpus).

Meta-analysis: Meta-analysis was performed using two different approaches. The first approach compared the effect sizes (ORs (95% CI)) for site-specific cancer risk of the highest category of dietary nitrate and nitrite against the lowest (reference) category. were compared. The second approach pooled the effect sizes (ORs (95% CI)) for site-specific cancer risk in all intake categories and compared it against the lowest (reference) category. When I2 statistics do not present a notable heterogeneity (*p*  >  0.05 or I2 ≤  50%), we used a fixed-effects analysis described by Mantel-Haenszel [49]; otherwise, a random-effects analysis was conducted described by the DerSimonian and Laird method [50]. Any cancer site that had less than three studies was not included in the meta-analysis. Two types of cancers with similar sites or very close locations in the human body were merged for analysis (these included ovarian cancer and uterus corpus). 

## 3. Results

### 3.1. Selection of the Studies 

**Figure 1:**
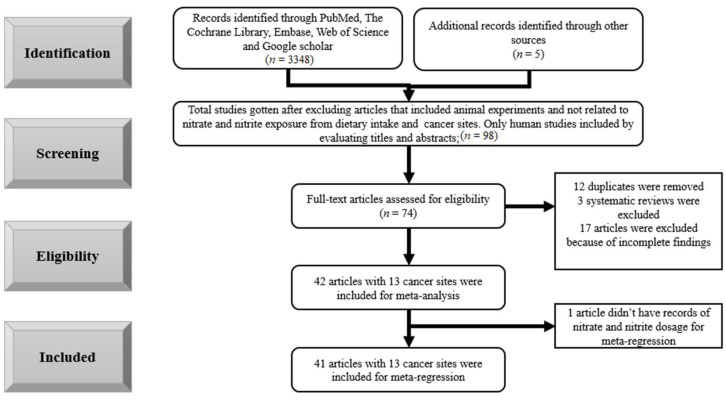
Flow chart of study selection.

A total of 3348 records were identified through database searching. After screening titles and abstracts according to the inclusion and exclusion criteria, 98 records remained. The full texts of 74 articles were assessed for eligibility, upon which 31 articles were excluded. Therefore, 41 articles were eligible for meta-regression with 13 cancer sites. The process of selection of studies can be seen in Figure 1. 

### 3.2. Results 

#### 3.2.1. Meta-Regression

**Figure 2:**
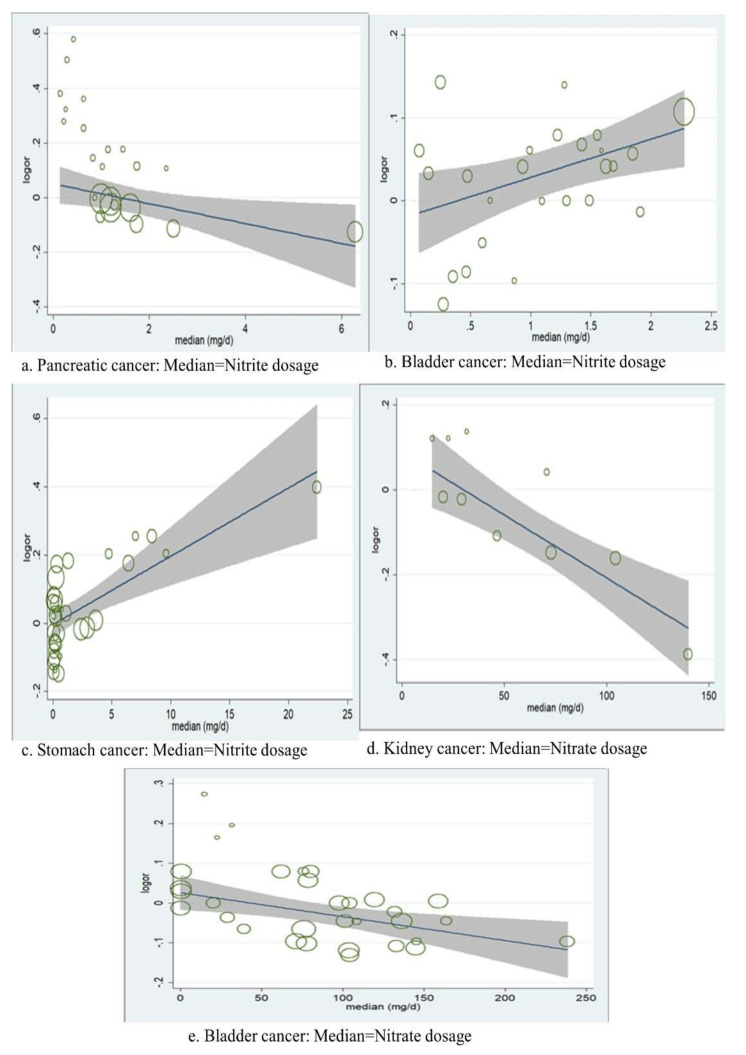
Meta-regression: the association between the risk of logarithm ORs and median dosage of dietary nitrite and nitrate for selected site-specific cancers; (**a**). Pancreatic cancer Median Nitrite dosage, (**b**). Bladder cancer: Median = Nitrite dosage, (**c**). Stomach cancer: Median = Nitrite dosage, (**d**). Kidney cancer: Median = Nitrate dosage, (**e**). Bladder cancer: Median = Nitrate dosage.

Meta-regression analyses showed that the risk of both bladder (t = 1.99, *p* = 0.056, adjusted R^2^ = 33.77%) and stomach cancers (t = 4.09, *p* = 0.000, adjusted R^2^= 74.06%) were positively associated with the dosage of dietary nitrite (Figure 2b,c, but that the risk of pancreatic cancer was inversely associated (t = −2.89, *p* = 0.007, adjusted R^2^ = 33.37%) (Figure 2a. In relation to the dosage of dietary nitrate, the risk of both kidney (t = −4.02, *p* = 0.002, adjusted R^2^ = 100%) and bladder cancers (t = −2.78, *p* = 0.008, and R^2^ =58.38%) were inversely associated (Figure 2d,e). No other significant associations were observed by meta-regression analyses. 

#### 3.2.2. Meta-Analysis

**Figure 3:**
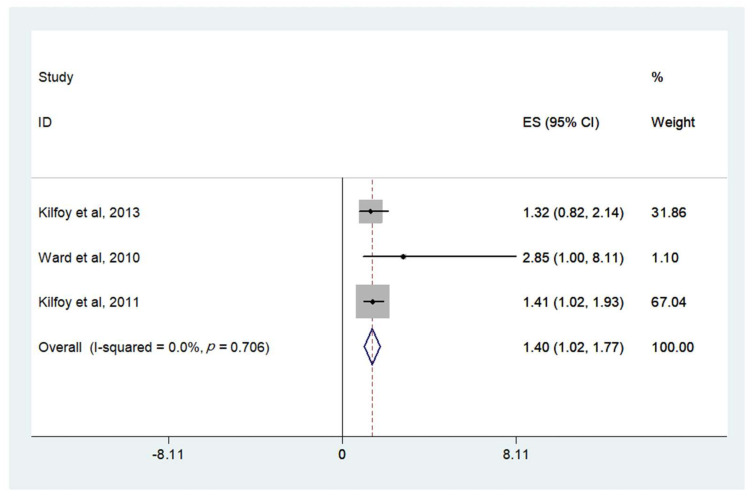
ORs (95% CI) for thyroid cancer of the highest versus lowest category of dosage of dietary nitrate consumption for the following selected studies .

When comparing highest to lowest (reference) categories of intake, meta-analysis of studies showed that high nitrate intake was associated with an increased risk of thyroid cancer (OR = 1.40, 95% CI: 1.02, 1.77) (Figure 3 and Table 1). Little heterogeneity was observed (I2 = 0.0%, *p* = 0.706). There was no evidence of association between the risk of cancers of the reproductive organs (ovary and uterine corpus), breast, non-Hodgkin’s lymphoma, stomach, pancreatic, esophageal, bladder, kidney, colon, or rectal cancer and dietary nitrate and nitrite (Table 1). 

**Figure 4:**
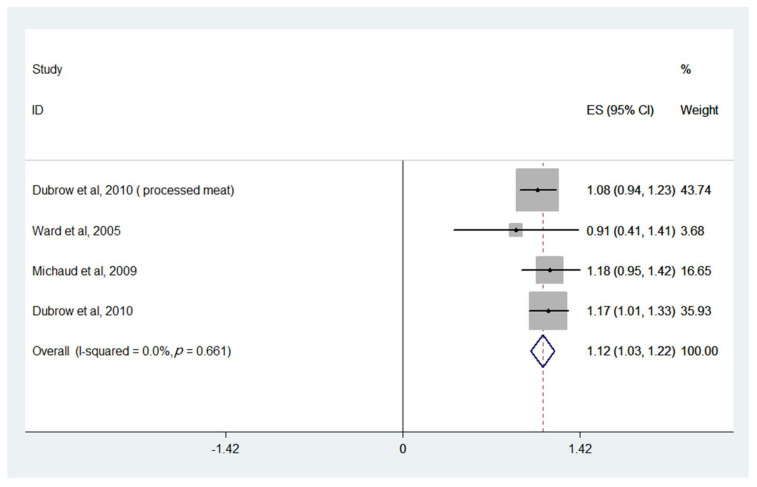
ORs (95% CI) for glioma of all combined higher dosages versus the lowest category of dietary nitrite consumption for the following selected studies.

When pooling all intake categories and comparing against the lowest (reference) category, higher nitrite intake was associated with an increased risk of glioma (OR = 1.12, 95% CI: 1.03, 1.22) (Figure 4 and Table 1). Little heterogeneity was observed (I2 = 0.0%, *p* = 0.661). There was no evidence of association between the risk of cancers of the reproductive organs (ovary and uterine corpus), breast, non-Hodgkin’s lymphoma, stomach, pancreatic, esophageal, bladder, kidney, colon and rectal cancer, and dietary nitrate or nitrite consumption.

All results are shown in Table 1, and the remaining figures are shown in the Supplementary Figures S1 and S2. 

#### 3.2.3. Publication Bias

**Figure 5:**
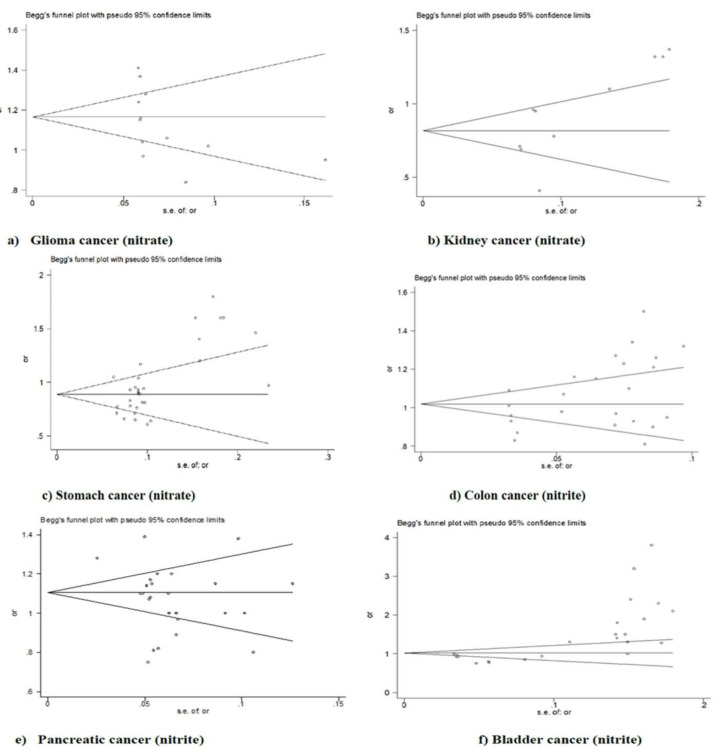
Funnel plot of nitrates and (**a**) glioma, (**b**) kidney, and (**c**) stomach cancer risk; nitrites and (**d**) colon, (**e**) pancreatic and (**f**) bladder cancer risk for publication bias.

Both the Egger and Begg tests of bias indicated asymmetry (publication bias) for both cancer of the kidney (Egger, *p* = 0.019; Begg, *p* = 0.016; Figure 5b and Table 2), and stomach (Egger, *p* = 0.000; Begg, *p* = 0.006; Figure 5c and Table 2) with nitrates, and pancreatic cancer with nitrites (Egger, *p* = 0.000; Begg, *p* = 0.000; Figure 5e and Table 2). The Egger’s test showed statistical evidence of bias for colon cancer and nitrites (*p* = 0.027; Figure 5d and Table 2) but the Begg’s test did not (*p* = 0.141). The Begg’s test showed statistical evidence of bias for glioma and nitrates (*p* = 0.040; Figure 5a and Table 2) but the Egger’s test did not (*p* = 0.132). No other evidence of bias was indicated (Table 3). The remaining figures are shown in the Supplementary Figure S3. 

## 4. Discussion

This systematic literature review and meta-analysis aimed to quantify the associations between cancer risk and dose of dietary nitrate and nitrite reported in the literature to date. Using 41 eligible articles, we conducted meta-analyses using two different approaches to compare the risk of 13 different site-specific cancers across different categories of dietary intake. Moreover, we conducted a meta-regression analysis to examine associations between site-specific cancer risk and dosage of dietary nitrates and nitrites. 

Firstly, when comparing highest to lowest (reference) categories of intake, meta-analysis showed that high nitrate intake was associated with an increased risk of thyroid cancer (OR = 1.40, 95% CI: 1.02, 1.77). When pooling all intake categories and comparing against the lowest (reference) category, higher nitrite intake was associated with an increased risk of glioma (OR = 1.12, 95% CI: 1.03, 1.22). 

Meta-regression analysis showed that bladder and stomach cancer risk was greater, and that pancreatic cancer risk was lower, with increasing nitrite intakes. Kidney and bladder cancer risk were both lower with increasing nitrate intakes. No other associations between cancer risk and dietary intakes of nitrates or nitrites were observed. These data suggest type- and site-specific effects on cancer risk, including protective effects, from dietary intakes of nitrate and nitrite. 

These findings from a meta-analysis of the literature are an important contribution, as individual studies on their own have reported seemingly inconsistent findings. Some studies have shown positive, and others, negative associations with cancer risk at different intakes of dietary nitrate and nitrite. For example, Kilfoya et al. [51] reported an association between ovarian cancer and a daily nitrate intake of 175.4 mg/day, HR: 1.31 (95% CI: 1.01, 1.68) in a 10-year prospective cohort study of women (aged 50–71 years), with a total of 709 incident epithelial ovarian cancer cases. This same study did not show any association between ovarian cancer and total nitrate intake, yet there was a relationship between a nitrate intake of 0.33 mg/1000 kcal from animal sources HR: 1.34 (95% CI: 1.05, 1.69). In contrast, Inoue-Choi et al., [5] did not show any association with the same range of nitrate daily intake 165.48–209.2 mg/day, HR: 0.85 (95% CI: 0.56, 1.27) in a similar cohort study of women aged 55–69 years. This is a good comparison because these two studies have almost the same daily nitrate intake and the same demographic characteristics, which is sometimes difficult to find. Three studies on breast cancer did not show any associations [12,52,53]. More research is needed to study the association between nitrate and nitrite intake and breast cancer from both food and water, especially since it is the second leading cause of cancer death in 92% of women. Non-Hodgkin’s lymphoma studies showed an association between daily nitrite intake and the disease [25,56,60]. Most studies had daily intake ranges of 0–2 mg/day; one study did not report the daily intake [25]. There was no association between daily nitrate intake and this cancer. Stomach cancer was the most studied cancer among the articles retrieved for this systematic review, yet only three of these studies showed any relationship with dietary nitrite intake. This study’s meta-regression showed an association between dietary nitrite exposure and stomach cancer. Daily nitrate intake was not associated with stomach cancer in the meta-regression analysis. As for other cancers (cancer of the colon, rectum, esophagus, pancreas, kidney, thyroid, and glioma), one or two studies showed positive associations with dietary nitrate and nitrite intake. 

Many studies have shown that a long period of exposure/daily dietary intake that contains nitrate, nitrite, and NOC compounds can lead to specific health issues, but these results are still contradictory. Keszei, et al. (2013) [69] conducted a cohort study with 16.3 years of follow-up in the Netherlands, from 1986 to 2002 for men and women aged 55–69 years. In this study, esophageal squamous cell carcinoma (ESCC) risk was associated with nitrite intake (HR for 0.1-mg/day increase: 1.19; 95% CI: 1.05, 1.36; *p*-trend = 0.06). Positive associations were observed between N-nitrosodimethylamine intake and esophageal squamous cell carcinoma (ESCC) risk (HR for 0.1 micro gram/day increase in intake: 1.15; 95% CI: 1.05, 1.25; *p*-trend = 0.01 based on tertiles of intake) and gastric non-cardia adenocarcinoma (GNCA) risk (1.06; 95% CI: 1.01, 1.10; *p*-trend = 0.09) in men. Meanwhile Cross et al., 2011, [53] conducted a cohort study with 10 years of follow-up in the USA, from 1995 to 2006 for men and women aged 50–71 years, which showed nitrate and nitrite were not associated with esophageal or gastric cancer. Some case-control studies and ecological studies have yielded inconclusive results about different types of cancers. 

Individually, most of the research articles included in this systematic review did not find any association between nitrate and nitrite and any type of cancer in humans. However, when analyzed together, greater exposure to dietary nitrate and nitrite increased the risk of getting some cancers (glioma, bladder, stomach, and thyroid) and decreased the risk of getting others (pancreatic and kidney). Most of these cancers can be considered cancers of the digestive system. The risk of these types of cancers have been shown to be modified by other dietary and lifestyle factors. For example, some studies have shown an inverse association of vegetable and fruit intake with these cancers’ risk [78]. Other studies have shown that people who take in high vitamin C, high vitamin E, low red meat (or any type of meat), and folate while being exposed to nitrate or nitrite at the same time had a lower risk of having cancer, than those who did not [64,79]. However, not all studies showed these protective effects. Zeegers et al., 2006, [65] showed that vitamin C (*p* = 0.63) and vitamin E (*p* = 0.62), did not appear to be significant effect modifiers in the association between nitrate exposure from food and bladder cancer risk. Catsburg et al., (2014) [38] showed that among individuals with high nitrate intake, a positive association between high (i.e., above the median) heme intake and risk of bladder cancer was observed (highest category vs. lowest category OR = 1.76; 95% CI = 1.21–2.55; *p* trend = 0.007). Some studies showed no association at all [33,67,80]. 

Notwithstanding the null findings of some studies, it is widely accepted that it is important that people consume diets high in fresh fruits and vegetables that contain a lot of vitamins and essential minerals and reduce meat, fatty food, and processed food intake to improve their health. This may be important in modifying any harmful effects of dietary nitrates and nitrites on particularly susceptible tissues in the digestive system and elsewhere in the body. Cases of cancer are considered to be linked to nutritional factors. Scientific evidence suggests that food/diet is most convincingly linked to cancer of the lung, stomach, rectum, colon, pharynx, nasopharynx, esophagus, and mouth [81,82,83]. Filtration and purification of drinking water from both private and public sources before consumption is extremely important because several studies have shown that the consumption of nitrate and nitrite from drinking water, even in a very small amount, over a long period can lead to cancer (a chronic disease) and other health issues [31,84,85]. 

Most of the studies included in this systematic review were conducted in Europe and the U.S., and very few or no studies from South America, Africa, Australia, and Asia were retrieved. Many studies and awareness of early screening for different types of cancers are needed in South America, Africa, Australia, and Asia to better understand this issue on a larger scale with different demographics. A proper and comprehensive assessment of nitrate and nitrite from dietary intake, including inhibitors of endogenous nitrosation and intakes of antioxidants, are needed in future studies. Many studies lacked information about study participants’ water consumption and dietary intake that contain nitrate and nitrite simultaneously, which may be essential to better analyze the links between dietary intakes and cancers. Future studies should also pay close attention to the different duration or lengths (years) of food intake with nitrite and nitrate, especially to understand the effects of exposure. There is still no precise standard maximum contaminant level for nitrate and nitrite in food to protect people from non-communicable diseases like cancer. This might be because noncommunicable diseases, such as cancer, can take a long time to occur and the casualty has not yet been fully established. 

### Limitations 

Using two different statistical analyses for each cancer site is the strength of this research. It can help to better understand the robustness of the associations. However, the study’s limitations are as follows; first, very few articles were available for each type of cancer, some with three or fewer studies, and so the results for this analysis should be treated with caution. More detailed, well-designed studies with accurate and precise information about study participants’ food intake and other lifestyle factors may be essential to more accurately estimate associated risk. Secondly, this research was unable to adjust for potential confounders or examine effect modification (e.g., of dietary vitamin C intake) due to lack of available data in the included studies. Some studies included vitamin C, D, and E, and folate and red/processed meat intake; however, most did not. We recommend that such research should include dietary vitamin C, D, and E, intake, as well as folate intake, polyphenols, red/processed meat, heme iron intake, and other nutrients/minerals and compounds (especially the dosage) from food and drinking water that could affect nitrosation in the body, to enable more precise estimation of risks and more sophisticated analyses.

Third, there was a wide range of nitrate and nitrite intake values from different studies, which resulted in different ranges across the analyses performed in this meta-analysis. For example, dietary nitrite ranged from 0 to 2.4 mg/day in the analysis against bladder cancer risk and from 0 to 22 mg/day in the analysis against stomach cancer risk. This variation in range in the independent variable across different analyses could be partly responsible for the variation in association with the dependent variables (the different site-specific cancer risks) observed in this study. A lack of standardized units for reporting dietary intakes of nitrates and nitrites in the literature made it necessary to convert the data to a common unit for meta-regression. Whilst this is not a major limitation, it has the potential to introduce error. Moreover, a lack of standardized units might pose a problem to efforts to implement a limit or precise standard maximum contaminant level to protect people from health risks of having a type of cancer. Lastly, many cohort and case-control studies should be done in different parts of the world to understand this topic better, especially noting other confounding factors and nutrient intake.

## 5. Conclusions 

This study showed varied associations between site-specific cancer risks and dietary intakes of nitrate and nitrite. Glioma, bladder, and stomach cancer risks were higher, but pancreatic cancer risk was lower with higher nitrite intakes. Thyroid cancer risk was higher, but kidney and bladder cancer risks were lower with higher nitrate intakes. These data suggest type- and site-specific effects of cancer risk, including protective effects, from dietary intakes of nitrate and nitrite.

## Figures and Tables

**Table 1:** Meta-analysis of pooled ORs (95% CI) of the highest versus lowest category and all combined higher versus the lowest category of dietary nitrate and nitrite consumption for selected, site-specific cancers.

Type of Cancer	Highest versus the Lowest (Reference) Category		All Combined Highest versus the Lowest (Reference) Category		Publication Bias	
	Pooled OR (95% CI)	I-Squared (*I^2^*) and *p*-Value	Pooled OR (95% CI)	I-Squared (*I^2^*) and *p*-Value	Egger’s Test *p*-Value	Begg’s Test *p*-Value
(a) Ovarian and uterine corpus (nitrate)	1.03, (0.84, 1.22)	28.7%, *p* = 0.240	0.97, (0.75, 1.19)	80.6%, *p* = 0.001	0.067	0.090
(b) Breast (nitrate)	0.91, (0.81, 1.00)	0.0%, *p* = 0.526	0.92, (0.87, 0.96)	42.7%, *p* = 0.175	0.310	0.144
(c) Thyroid (nitrate)	1.40, (1.02, 1.77)	0.0%, *p* = 0.706	1.27, (0.85, 1.69)	62.0%, *p* = 0.072	0.064	0.325
(d) Glioma (nitrate)	1.11, (0.91, 1.31)	0.0%, *p* = 0.546	1.11, (0.94, 1.29)	66.9%, *p* = 0.049	0.132	0.040
(e) Glioma (nitrite)	1.17, (0.98, 1.37)	0.0%, *p* = 0.646	1.12, (1.03, 1.22)	0.0%, *p* = 0.661	0.442	0.060
(f) Non-Hodgkin’s Lymphoma (nitrate)	0.82, (0.69, 0.94)	27.1%, *p* = 0.195	0.83, (0.75, 0.91)	35.6%, *p* = 0.124	0.163	0.728
(g) Non-Hodgkin’s Lymphoma (nitrite)	1.21, (0.78, 1.64)	63%, *p* = 0.019	1.11, (0.85, 1.38)	71.4%, *p* = 0.004	0.496	0.702
(h) Pancreatic (nitrate)	0.96, (0.84, 1.09)	35.9%, *p* = 0.167	0.95, (0.89, 1.00)	48.0%, *p* = 0.087	0.722	0.399
(i) Pancreatic (nitrite)	0.87, (0.76, 0.97)	44.3%, *p* = 0.095	1.04, (0.85, 1.24)	76.1%, *p* = 0.000	0.000	0.000
(j) Bladder (nitrate)	0.94, (0.84, 1.04)	0.0%, *p* = 0.615	0.94, (0.84, 1.03)	70.7%, *p* = 0.001	0.089	0.322
(k) Bladder (nitrite)	1.07, (0.94, 1.19)	0.0%, *p* = 0.574	1.05, (0.92, 1.18)	79.2%, *p* = 0.000	0.045	0.338
(l) Kidney (nitrate)	0.79, (0.17, 1.41)	64.7%, *p* = 0.059	0.84, (0.52, 1.16)	73.1%, *p* = 0.024	0.019	0.016
(m) Kidney (nitrite)	0.92, (0.62, 1.23)	0.0%, *p* = 0.586	1.10, (0.78, 1.48)	76.7%, *p* = 0.014	0.322	0.245
(n) Colon (nitrate)	0.99, (0.91, 1.08)	40.1%, *p* = 0.100	1.00, (0.96, 1.04)	0.0%, *p* = 0.593	0.332	0.284
(o) Colon (nitrite)	1.02, (0.92, 1.11)	44.8%, *p* = 0.093	1.02, (0.93, 1.11)	67.2%, *p* = 0.006	0.027	0.141
(p) Rectal (nitrate)	1.01, (0.88, 1.14)	33.4%, *p* = 0.161	1.10, (0.96, 1.24)	68.8%, *p* = 0.002	0.367	0.930
(q) Rectal (nitrite)	1.09, (0.79, 1.39)	68.2%, *p* = 0.008	1.06, (0.87, 1.26)	82.3%, *p* = 0.000	0.841	0.952
(r) Esophageal (nitrate)	0.75, (0.57, 0.94)	46.1%, *p* = 0.073	0.83, (0.72, 0.94)	33.7%, *p* = 0.159	0.983	0.446
(s) Esophageal (nitrite)	1.01, (0.78, 1.23)	0.0%, *p* = 0.689	0.93, (0.81, 1.05)	0.0%, *p* = 0.881	0.197	0.714
(t) Stomach (nitrate)	0.81, (0.70, 0.92)	0.0%, *p* = 0.776	0.81, (0.75, 0.87)	22.0%, *p* = 0.234	0.000	0.006
(u) Stomach (nitrite)	1.06, (0.92, 1.20)	32.9%, *p* = 0.127	1.04, (0.91, 1.11)	54.7%, *p* = 0.012	0.308	0.382

I-squared (*I^2^*), a statistic representing the amount of total variation attributed to heterogeneity; *p*-value of Cochran’s Q test for heterogeneity.

**Table 2:** Characteristics of the included studies and reported associations between dietary nitrate (mg/day) and cancer risk.

First Author, Year, Country	Study Design	Case	Control/Number of Person-Years	Exposure Categories Nitrate Intake (mg/day)	Reported OR/RR/HR 95 CI	Cancer Sites	Adjustment	NOS
Briseis Aschebrook-Kilfoya et al., 2012, USA [51]	Cohort study, 1995–1996	128 143 143 140 155	⁕	36.7 58.1 78.4 109.5 175.4	1 (Reference) 1.13 (0.89–1.44) 1.15 (0.9–1.44) 1.14 (0.89–1.46) 1.31 (1.01–1.68)	Ovary	Age, race, total energy intake, family history of ovarian cancer, BMI, education, smoking status, menopausal status, parity, age at menarche, and total daily dietary vitamin C intake	8
Peter J. Weyer et al., 2001, USA [52]	Cohort study, 1986–1998	24 28 28 22	⁕	0–11.6 11.6–18 18.1–27.2 27.2–36.3	1 (Reference) 1.12 (0.65–1.94) 1.1 (0.63–1.92) 0.85 (0.47–1.55)	Ovary	Age and total energy intake	7
		71 41 51 61	⁕	0–11.6 11.6–18 18.1–27.2 27.2–36.3	1 (Reference) 0.6 (0.41–0.88) 0.78 (0.54–1.12) 0.97 (0.68–1.39)	Uterine corpus		
Maki Inoue-Choi et al., 2015, USA [12]	Population-based cohort, 1986–2010	59 73 54 74 55	⁕	3.87–65.43 65.44–92.04 92.05–121.96 121.97–165.48 165.48–209.2	1 (Reference) 1.18 (0.83–1.68) 0.86 (0.58–1.26) 1.21 (0.84–1.74) 0.85 (0.56–1.27)	Ovary	Age, BMI, family history of ovarian cancer, number of live births, age at menarche, age at menopause, age at first live birth, oral contraceptive use, estrogen use, and history of unilateral oophorectomy, and total energy intake	8
Maki Inoue-Choi et al., 2012, USA [26]	Prospective cohort study, 1986–2008	604 541 575 601 554	⁕	3.9–65.2 65.2–91.8 91.8–121.8 121.8–165.6 165.6–209.9	1 (Reference) 0.86 (0.76–0.98) 0.9 (0.79–1.02) 0.96 (0.84–1.1) 0.86 (0.74–1.01)	Breast	Age, total energy intake, BMI, WHR, education, smoking, physical activity level, alcohol intake, family history of breast cancer, education, smoking status, age at menopause, age at first live birth, estrogen use, total intake of folate, vitamin C and E intake and flavonoids, intake of cruciferae and red meat	8
Nadia Espejo-Herrera et al., 2016, Spain [53]	Multicase–Control Study, 2008–2013	387 349 348	⁕	0–90 90–138 138–186	1 (Reference) 0.9 (0.74–1.1) 0.9 (0.73–1.1)	Breast	Study area, age, and education	6
Peter J. Weyer et al., 2001, USA [52]	Cohort study in Iowa, 1986–1998	253 252 265 254	⁕	0–11.6 11.6–18 18.1–27.2 27.2–36.3	1 (Reference) 0.98 (0.83–1.17) 1.04 (0.87–1.24) 0.99 (0.83–1.19)	Breast	Age and total energy intake	7
Briseis Aschebrook-Kilfoy, et al., 2013, China [14]	Cohort study, 1996–2009	34 56 41 33	⁕	165.8 257.8 350.6 506.8	1 (Reference) 1.81 (1.18–2.76) 1.44 (0.92–2.28) 1.32 (0.82–2.14)	Thyroid	Age, total energy intake, education, and history of thyroid disease	8
Mary H. Ward, et al., 2010, USA [54]	Cohort study, 1986–2004	6 10 10 14	77,806 86,270 89,707 83,454	0–17.4 17.5–27.7 27.8–41.1 41.1–54.4	1 (Reference) 1.65 (0.59–4.61) 1.69 (0.58–4.84) 2.85 (1–8.11)	Thyroid	Age, total calories, vitamin C intake, and residence location	8
Briseis Aschebrook-Kilfoy et al., 2011, USA [55]	Prospective cohort study, 1995–2003	63 67 60 74 106	⁕	29.6 49.8 70.2 100.9 166.8	1 (Reference) 1.01 (0.72–1.43) 0.87 (0.61–1.24) 1.04 (0.74–1.45) 1.41 (1.02–1.93)	Thyroid	Age	8
Dominique S. Michaud, et al. 2009, USA [32]	3 prospective cohort studies, 1976–2005	67 74 60 59 75	Sub-cohort (PY) =815,155 833,168 811,541 822,304 818,945	⁑ 69.3 94.7 127.7 180	1 (Reference) 1.06 (0.76–1.48) 0.84 (0.57–1.22) 0.95 (0.46–1.98) 1.02 (0.66–1.58)	Glioma	Age and caloric intake	6
*Robert Dubrow et al., 2010, USA [29]	Prospective cohort study, 1995–2003	98 114 135 126 112	⁕	48.38 74.8 102.38 143.5 237.13	1 (Reference) 1.16 (0.89–1.52) 1.41 (1.09–1.84) 1.37 (1.05–1.79) 1.28 (0.97–1.7)	Glioma	Sex, age, race, energy intake, education, height, and history of cancer at baseline	8
		100 121 135 109 120	⁕	0.275 (nitrite plus nitrate) 0.725 1.225 1.925 3.575	1 (Reference) 1.15 (0.88–1.5) 1.24 (0.95–1.61) 0.97 (0.74–1.28) 1.04 (0.79–1.36)			
Mary H. Ward et al., 2006, USA [56]	Case-control study, 1998–2000	156 116 111 80	98 98 98 97	0–76 76–113.9 114–169.9 170–225.9	1 (Reference) 0.75 (0.51–1.1) 0.71 (0.47–1.07) 0.54 (0.34–0.86)	Non-Hodgkin’s lymphoma	Age, education, sex, study center, race, dietary vitamin C, and total energy	7
Mary H. Ward, et al., 1996, USA [57]	Case-control study, 1950–1987	35 38 20 11	82 106 86 64	0–13 13–19 19–26 26–33	1 (Reference) 1.1 (0.6–2.0) 0.8 (0.4–1.7) 0.7 (0.3–1.9)	Non-Hodgkin’s lymphoma	Age, gender, family history of cancer, vitamin C, and carotenes	7
Peter J. Weyer, et al., 2001, USA [52]	Cohort study, 1986–1998	37 34 25 38	⁕	0–11.6 11.6–18 18.1–27.2 27.2–36.3	1 (Reference) 0.88 (0.55–1.4) 0.62 (0.37–1.04) 0.91 (0.56–1.46)	Non-Hodgkin’s lymphoma	Age and total energy intake	7
Briseis Aschebrook-Kilfoya et al., 2012, USA [58]	Case-control, 1996–2008	⁕	⁕	0–62.8 62.8–95.9 95.9–141 141–186.1	1 (Reference) 1 (0.7–1.4) 1.1 (0.7–1.6) 1 (0.7–1.5)	Non-Hodgkin’s lymphoma	Calories, age, family history, and vitamin C	7
Briseis Aschebrook-Kilfoya et al., 2013, USA [59]	Case-control, 1999–2002	100 83 80 72	115 115 114 115	46.5 80.5 110.9 178.5	1 (Reference) 0.9 (0.6–1.3) 0.9 (0.6–1.3) 0.8 (0.5–1.3)	Non-Hodgkin’s lymphoma	Sex, age, body mass index, education, family history of cancer, vitamin C, and daily caloric intake	6
Brian C.-H. Chiu et al., 2008, USA [60]	Case-control study, 1983–1986	17 19 24	357 358 360	0–70 70–106 106–142	1 (Reference) 1 (0.5–1.9) 1.2 (0.6–2.4)	Non-Hodgkin’s lymphoma	Age, sex, type of respondent, family history of cancer, and body mass index	8
		17 19 24	357 358 360	0–65 65–101 101–137	1 (Reference) 1 (0.5–1.9) 1.2 (0.6–2.4)			
		36 28 23	357 358 360	0–70 70–106 106–142	1 (Reference) 0.8 (0.5–1.3) 0.7 (0.4–1.2)			
		36 29 22	357 358 360	0–65 65–101 101–137	1 (Reference) 0.8 (0.5–1.3) 0.6 (0.3–1.1)			
Briseis Aschebrook-Kilfoya et al., 2010, USA [25]	Case-control study, 1995–2001	274 317	352 355	Low High	1 (Reference) 1.09 (0.86–1.39)	Non-Hodgkin’s lymphoma	Age, family history of cancer, calories, vitamin C intake, vitamin E intake, and protein intake	7
Arbor J.L. Quist et al., 2018, USA [61]	Cohort study, 1986–2011	78 80 73 60 17	*n* = 8558 8552 8568 6849 1715	0–16.2 16.2–23.9 24–34.2 34.3–58.5 58.5–82.7	1 (Reference) 1.08 (0.78–1.48) 0.99 (0.7–1.39) 1.05 (0.72–1.52) 1.25 (0.71–2.21)	Pancreas	Age, smoking category, calories, and mutually adjusted for either natural log-transported nitrate or nitrite	
Angela Coss, et al., 2004, USA [62]	Case-control study, 1960–1987	26 33 39 43	298 311 311 327	0–58 58–82 83–117 117–151	1 (Reference) 1.1 (0.63–1.9) 1.2 (0.7–2) 1 (0.6–1.8)	Pancreas	Age, cigarette use, and caloric intake	
		39 33 24 26	164 157 158 160	0–63 63–90 91–126 126–161	1 (Reference) 0.99 (0.58–1.7) 0.64 (0.36–1.1) 0.53 (0.29–0.97)			
Peter J. Weyer et al., 2001, USA [52]	Cohort study, 1986–1998	19 15 16 19	⁕	0–11.6 11.6–18 18.1–27.2 27.2–36.3	1 (Reference) 0.79 (0.4–1.56) 0.86 (0.44–1.69) 1.02 (0.52–1.99)	Pancreas	Age and total energy intake	7
*Jiali Zheng et al., 2019, USA [63]	Case–control study, 2002–2009	283 236 192 271	235 234 235 234	9.18–73.5 69.4–101.1 92.8–133.6 119.1–715.9	1 (Reference) 0.93 (0.72–1.2) 0.76 (0.59–0.99) 1.08 (0.84–1.39)	Pancreas	Age and energy intake	
Briseis Aschebrook-Kilfoy et al., 2011, USA [64]	Prospective cohort study, 1995–2006	370 330 360 340 322	⁕	34.8 56.9 75.0 95.3 150.3	1 (Reference) 0.91 (0.78–1.06) 1.02 (0.88–1.18) 0.99 (0.85–1.16) 1.01 (0.85–1.2)	Pancreas	Age, race, total energy intake, smoking status, family history of cancer, family history of diabetes, body mass index, and intakes of saturated fat, folate, and vitamin C	
Rena R. Jones, et al., 2016, USA [8]	Cohort study, 1986–2010	67 68 64 59	*n* = 8467 8489 8, 506 8502	0–16.2 16.2–23.9 24–34.2 34.2–44.4	1 (Reference) 1 (0.72–1.41) 0.92 (0.66–1.3) 0.86 (0.6–1.22)	Bladder	Age and total in-transformed dietary nitrite from all sources	
Maurice P. Zeegers et al., 2006, Netherlands [65]	Cohort study, 1986–1995	168 186 181 180 174	Subcohort (PY) =8512 8652 8706 8707 8564	2–69 69–88 88–107.5 107.5–135.3 135.3–451.1	1 (Reference) 1.14 (0.89–1.45) 1 (0.78–1.27) 1.02 (0.8–1.3) 1.01 (0.79–1.29)	Bladder	Age and sex	
Chelsea E. Catsburg et al., 2014, USA [38]	Case-control study, 1987–1996	467 329 293 274 284	315 314 315 315 314	0–64.3 64.4–91.4 91.5–117.3 117.4–148.3 148.4–179.3	1 (Reference) 0.79 (0.63–1.01) 0.74 (0.57–0.97) 0.78 (0.58–1.06) 0.9 (0.6–1.35)	Bladder	Smoking duration, smoking intensity, and smoking status	8
Mary H. Ward et al., 2003, USA [35]	Case-control study, 1986–1989.	⁕	⁕	0–59 59–84 84–119 119–154	1 (Reference) 0.8 (0.7–1.1) 0.9 (0.7–1.2) 0.9 (0.7–1.1)	Bladder	Age, education, and cigarette smoking, years chlorinated surface water, and study period	
		⁕	⁕	0–62 62–90 90–127 127–164	1 (Reference) 1.2 (0.8–1.9) 0.9 (0.5–1.4) 0.8 (0.5–1.3)			
Peter J. Weyer, et al., 2001, USA [52]	Cohort study, 1986–1998	9 17 13 14	⁕	0–11.6 11.6–18 18.1–27.2 27.2–36.3	1 (Reference) 1.88 (0.84–4.24) 1.46 (0.62–3.47) 1.57 (0.66–3.75)	Bladder	Age and total energy intake	7
Kathryn Hughes Barry et al., 2020, New England [66]	Case–control study, 1994–1996 and 2001–2004	227 230 225 183 172	247 245 243 246 244	0–21.9 21.19–28.28 28.28–36.10 36.10–47.21 >47.21	1 (Reference) 1.2 (0.88–1.5) 1.2 (0.92–1.6) 1.0 (0.75–1.4) 0.95 (0.69–1.3)	Bladder	Adjusted for age, gender, smoking status, high-risk occupation, race, ethnicity, state, dietary vitamin C intake (per 1000 kcal—continuous), dietary vitamin B12 (per 1000 kcal—continuous), total energy intake (kcal—continuous), and total water intake (L/d—continuous); models for nitrate/nitrite from processed meat were additionally adjusted for total meat intake (per 1000 kcal—continuous)	
*Leah M. Ferrucci et al., 2010, USA [5]	Cohort study, 1995–2003	236 185 150 145 138	⁕	49.25 76 103.75 145 238.5	1 (Reference) 0.86 (0.71–1.06) 0.76 (0.6–0.95) 0.77 (0.6–0.99) 0.8 (0.58–1.1)	Bladder	Age, gender, smoking, intakes of fruit, vegetables, beverages, and total energy	
		109 147 173 191 234	⁕	0.05 0.175 0.275 0.425 0.725	1 (Reference) 0.97 (0.76–1.24) 1.09 (0.87–1.38) 1.07 (0.85–1.36) 1.2 (0.95–1.51)			
Mary H. Ward et al., 2007, USA [27]	Case-control study, 1986–1989.	109 83 84 57	471 472 471 472	0–59.32 59.32–86.62 86.63–122 122–157.77	1 (Reference) 0.71 (0.52–0.98) 0.69 (0.5–0.95) 0.41 (0.28–0.6)	Kidney	Age, gender, sodium, and total calories	
Rena R. Jones et al., 2017, USA [67]	Cohort study, 1986–2010	67 65 66 43 15	*n* = 8467 8489 8, 506 6803 1699	0–16.2 16.2–23.9 23.91–34.27 34.28–58.64 58.6–82.96	1 (Reference) 0.96 (0.68–1.4) 0.95 (0.67–1.4) 0.78 (0.51–1.2) 1.1 (0.59–2)	Kidney	Age, smoking status, pack-years of smoking, in-transformed total energy intake, body mass index, and total in-transformed total dietary nitrate or nitrite	
Peter J. Weyer et al., 2001, USA [52]	Cohort study from 1986–1998	12 15 14 14	⁕	0–11.6 11.6–18 18.1–27.2 27.2–36.3	1 (Reference) 1.32 (0.62–2.83) 1.32 (0.6–2.89) 1.37 (0.61–3.06)	Kidney	Age and total energy intake	
Mary H. Ward et al., 2008, USA [68]	Case-control study from 1988–1994	14 17 28 39	99 99 99 100	0–3.8 3.8–5.7 5.7–8.3 8.3–10.9	1 (Reference) 0.7 (0.3–1.6) 1.7 (0.7–4.1) 2.2 (0.9–5.7)	Esophagus	Year of birth, gender, body mass index, smoking, alcohol, total calories, vitamin A, folate, riboflavin, zinc, protein, and carbohydrate	
		29 27 18 24	99 99 99 100	0–16.9 16.9–26.2 26.2–38.8 38.8–51.4	1 (Reference) 0.9 (0.5–1.8) 0.6 (0.3–1.3) 0.8 (0.3–1.8)			
Andra’s P. Keszei et al., 2013, The Netherlands [69]	Cohort study, 1986–2002	24 21 14	Sub-cohort (PY) 8383 9015 9050	68.1 100.8 146.2	1 (Reference) 0.82 (0.45–1.48) 0.54 (0.28–1.05)	Esophagus	Age	
		39 36 39	8383 9015 9050	68.1 100.8 146.2	1 (Reference) 0.86 (0.54–1.37) 0.92 (0.58–1.46)			
		15 18 15	9607 10,175 9996	66.4 98.5 142.7	1 (Reference) 1.17 (0.58–2.35) 1.02 (0.49–2.14)			
		14 13 4	9607 10,175 9996	66.4 98.5 142.7	1 (Reference) 0.89 (0.42–1.92) 0.29 (0.09–0.89)			
*Amanda J. Cross, et al., 2011, USA [24]	Cohort study, 1995–2006	22 25 15 25 41	⁕	0.605 0.1673 0.2818 0.4363 0.745	1 (Reference) 1.06 (0.59–1.91) 0.6 (0.3–1.18) 0.9 (0.49–1.67) 1.3 (0.72–2.35)	Esophagus	Age, sex, BMI, education, ethnicity, tobacco smoking, alcohol drinking, usual physical activity at work, vigorous physical activity, daily intake of fruit, vegetables, saturated fat, and calories	
		47 61 68 89 112	⁕	0.605 0.1673 0.2818 0.4363 0.745	1 (Reference) 0.97 (0.66–1.43) 0.91 (0.62–1.35) 1.01 (0.7–1.47) 1.1 (0.75–1.6)			
A. J. M. van Loon et al., 1998, The Netherlands [70]	Cohort study, 1986–1992	69 61 45 49 58	Sub-cohort (PY) 3784 3813 3814 3813 3796	55.8 79.4 98.7 120.7 172.2	1 (Reference) 0.93 (0.64–1.33) 0.65 (0.44–0.96) 0.71 (0.48–1.04) 0.83 (0.58–1.2)	Stomach	Age and sex	
Raúl U. Hernández-Ramírez et al., 2009, Mexico [71]	Case-control study, 2004–2005	⁕	⁕	0–90.4 90.4–141.7 141.7–193	1 (Reference) 0.93 (0.62–1.39) 0.61 (0.39–0.96)	Stomach	Energy, age, gender, *H. pylori* CagA status, schooling, and consumptions of salt, chili, and alcohol.	
Andra´s P. Keszei et al., 2013, The Netherlands [69]	Cohort study, 1986–2002	49 47 43	Sub-cohort (PY) 8383 9015 9050	68.1 100.8 146.2	1 (Reference) 0.89 (0.59–1.35) 0.81 (0.53–1.24)	Stomach	Age	
		111 125 93	8383 9015 9050	68.1 100.8 146.2	1 (Reference) 1.05 (0.79–1.39) 0.77 (0.57–1.04)			
		7 7 10	9607 10,175 9996	66.4 98.5 142.7	1 (Reference) 0.97 (0.34–2.81) 1.46 (0.54–3.93)			
		59 46 55	9607 10,175 9996	66.4 98.5 142.7	1 (Reference) 0.76 (0.51–1.13) 0.95 (0.64–1.4)			
Carlo La Vecchia, et al., 1994, Italy [72]	Case-control study, 1985–1992	⁕	⁕	62.95 80.7 96.33 116.88	1 (Reference) 0.71 (0.53–0.96) 0.66 (0.47–0.92) 0.78 (0.54–1.12)	Stomach	Age, sex, education, family history of gastric cancer, body mass index, total energy intake, plus all above variables	5
* Amanda J. Cross et al., 2011, USA [24]	Cohort study, 1995–2006	50 48 50 56 73	⁕	0.605 0.1673 0.2818 0.4363 0.745	1 (Reference) 0.9 (0.6–1.35) 0.89 (0.59–1.33) 0.91 (0.61–1.37) 1.04 (0.69–1.55)	Stomach	Age, sex, BMI, education, ethnicity, tobacco smoking, alcohol drinking, usual physical activity at work, vigorous physical activity, daily intake of fruit, vegetables, saturated fat, and calories.	
		39 57 36 61 62	⁕	0.605 0.1673 0.2818 0.4363 0.745	1 (Reference) 1.17 (0.77–1.77) 0.64 (0.4–1.02) 0.94 (0.61–1.45) 0.81 (0.52–1.25)			
Mary H. Ward, et al., 2008, USA [68]	Case-control study from 1988–1994	19 31 25 29	99 99 99 100	0–3.8 (nitrite plus nitrate) 3.8–5.7 5.7–8.3 8.3–10.9	1 (Reference) 1.6 (0.8–3.2) 1.8 (0.8–3.8) 1.6 (0.7–3.7)	Stomach	Year of birth, gender, education, smoking, alcohol, total calories, vitamin C, fiber, and carbohydrate.	
		24 28 26 26	99 99 99 100	0–16.9 16.9–26.2 26.2–38.8 38.8–51.4	1 (Reference) 1.2 (0.6–2.5) 1.4 (0.7–2.9) 1.6 (0.7–3.6)			
Curt T. Della Valle et al., 2014, China [44]	Prospective cohort study, 1996 to 2007	83 70 65 87 78	⁕	98.7 144.1 182.4 229 313.2	1 (Reference) 0.9 (0.65–1.25) 0.84 (0.59–1.2) 1.13 (0.77–1.66) 0.98 (0.59–1.63)	Colon	Age, energy intake, education, physical activity, dietary vitamin C intake, carotene, and folate	
Nadia Espejo-Herrera et al., 2016, Spain [53]	Case-control study, 2008–2013	388 394 371	⁕	0–83 83–133 133–183	1 (Reference) 1.04 (0.87–1.24) 0.9 (0.74–1.1)	Colon	Sex, age, education, physical activity, non-steroidal anti-inflammatory drugs use, family history of colorectal cancer, body mass index, and intake energy	
Rena R. Jones et al., 2019, USA [41]	Cohort study, 1986–2010	324 324 321 355	*n* = 8676 8674 8685 8673	0–9.8 9.81–13.8 13.81–19.29 19.29–24.77	1 (Reference) 0.98 (0.84–1.15) 0.97 (0.83–1.13) 1.11 (0.94–1.3)	Colon	Age, heme iron, red meat, and total dietary nitrate or nitrite	
Peter J. Weyer et al., 2001, USA [52]	Cohort study, 1986–1998	98 78 90 97	⁕	0–11.6 11.6–18 18.1–27.2 27.2–36.3	1 (Reference) 0.79 (0.59–1.07) 0.93 (0.69–1.24) 1 (0.74–1.34)	Colon	Age and total energy intake	7
* Amanda J. Cross et al., 2010, USA [29]	Prospective cohort study, 1994–2003	341 344 386 439 485	⁕	0.0598 0.1633 0.274 0.423 0.723	1 (Reference) 0.93 (0.8–1.08) 0.99 (0.86–1.16) 1.08 (0.93–1.25) 1.13 (0.97–1.32)	Colon	Gender, education, BMI, smoking, and intake of total energy, fiber, and dietary calcium	
* L. M. Ferrucci et al., 2012, USA [73]	Multi-center, randomized controlled trial, 1993–2001.	150 165 203 254	⁕	0.15 (nitrite plus nitrate) 0.425 0.9 2.1	1 (Reference) 0.98 (0.77–1.23) 1.07 (0.84–1.35) 1.16 (0.9–1.5)	Colon	Age, study center, gender, ethnicity, education, family history of colorectal cancer, BMI, NSAIDs use, physical activity, smoking status, alcohol intake, dietary calcium, supplemental calcium, dietary fibre, and total energy intake	
Anneclaire J. De Roos et al., 2003, USA [28]	Case-control study, 1986–1990	(*n*(%)) 89 (32) 68 (24) 68 (24) 55 (20)	(*n*(%)) 261 (27) 241 (25) 246 (25) 234 (24)	0–59.3 59.3–86.5 86.6–121.9 122–157	1 (Reference) 0.8 (0.6–1.2) 0.8 (0.5–1.1) 0.7 (0.4–1)	Colon	Age, sex, and chlorinated surface water	
Yun Zhu et al. 2014 [74]	Case-control study, 1997–2006	127 153 122 137 122	517 480 489 479 516	56.94 91.45 124.81 169.59 264.14	1 (Reference) 1.25 (0.93–1.66) 0.9 (0.66–1.23) 1.06 (0.78–1.48) 0.75 (0.54–1.05)	Colon	Age, sex, energy intake, BMI, cigarette smoking status, education attainment, reported colon screening procedures, NSAID use, multivitamin supplement use, folate supplement use, vegetable intake, and province of residence	
		109 113 128 122 114	517 480 489 479 516	56.94 91.45 124.81 169.59 264.14	1 (Reference) 1.07 (0.78–1.48) 1.24 (0.9–1.71) 1.31 (0.94–1.83) 1.01 (0.71–1.45)			
Curt T. Della Valle et al., 2014, China [44]	Prospective cohort study, 1996–2007	46 39 41 51 59	⁕	98.7 144.1 182.4 229 313.2	1 (Reference) 0.9 (0.58–1.4) 0.95 (0.6–1.5) 1.17 (0.72–1.9) 1.26 (0.69–2.32)	Rectum	Age, energy intake, education, physical activity, dietary vitamin C intake, carotene, and folate	
Nadia Espejo-Herrera et al., 2016, Spain [53]	Case-control study, 2008–2013.	195 161 151	⁕	0–83 83–133 133–183	1 (Reference) 0.85 (0.66–1.08) 0.76 (0.58–1)	Rectum	Sex, age, education, physical activity, non-steroidal anti-inflammatory drugs use, family history of colorectal cancer, body mass index, and intake energy	
Rena R. Jones et al., 2019, USA [41]	Cohort study, 1986–2010	79 81 71 94	*n* = 8676 8674 8685 8673	0–9.8 9.81–13.8 13.81–19.29 19.29–24.77	1 (Reference) 1.03 (0.76–1.41) 0.91 (0.66–1.26) 1.27 (0.93–1.74)	Rectum	Age and total dietary nitrate or nitrite	
Peter J. Weyer et al., 2001, USA [52]	Cohort study, 1986–1998.	28 39 27 28	⁕	0–11.6 11.6–18 18.1–27.2 27.2–36.3	1 (Reference) 1.42 (0.87–2.31) 1.01 (0.59–1.73) 1.06 (0.61–1.83)	Rectum	Age and total energy intake	7
* Amanda J. Cross et al., 2010, USA [29]	Prospective cohort study, 1994–2003	110 126 144 170 174	⁕	0.0598 0.1633 0.274 0.423 0.723	1 (Reference) 1.08 (0.83–1.4) 1.18 (0.91–1.52) 1.31 (1.01–1.68) 1.26 (0.97–1.63)	Rectum	Gender, education, BMI, smoking, and intake of total energy, fiber, and dietary calcium.	
* L. M. Ferrucci et al., 2012, USA [73]	Multi-center, randomized controlled trial, 1993–2001	44 64 75 80	⁕	0.15 (nitrite plus nitrate) 0.425 0.9 2.1	1 (Reference) 1.31 (0.88–1.95) 1.38 (0.92–2.07) 1.27 (0.8–1.99)	Rectum	Age, study center, gender, ethnicity, education, family history of colorectal cancer, BMI, NSAIDs use, physical activity, smoking status, alcohol intake, dietary calcium, supplemental calcium, dietary fiber, and total energy intake	
Anneclaire J. De Roos et al., 2003, USA [28]	Case-Control study, 1986–1990	(*n*(%)) 56 (22) 67 (27) 66 (27) 60 (24)	(*n*(%)) 261 (27) 241 (25) 246 (25) 234 (24)	0–59.3 59.3–86.5 86.6–121.9 122–157	1 (Reference) 1.3 (0.9–1.9) 1.2 (0.8–1.8) 1.1 (0.8–1.7)	Rectum	Age, sex, and chlorinated surface water	
Yun Zhu, et al. 2014 [74]	Case-control study, 1997–2006	118 126 130 133 118	517 480 489 479 516	56.94 91.45 124.81 169.59 264.14	1 (Reference) 1.12 (0.83–1.53) 1.23 (0.9–1.69) 1.34 (0.94–1.85) 1.03 (0.73–1.46)	Rectum	Age, sex, energy intake, BMI, cigarette smoking status, education attainment, reported colon screening procedures, NSAID use, multivitamin supplements use, folate supplement use, vegetable intakes and province of residence.	

* Original exposure categories of nitrates from studies converted to mg/day for meta-regression calculation (explained under statistics analysis); ⁕ missing cases/controls/person-years in sub-cohort from the studies; ⁑ missing nitrate dosage.

**Table 3:** Characteristics of the included studies and reported associations between dietary nitrite (mg/day) and cancer risk.

First Author, Year, Country	Study Design	Case	Control	Exposure Categories Nitrite intake mg/day)	Reported OR/RR/HR 95 CI	Cancer sites	Adjustment	NOS
* Robert Dubrow et al., 2010, USA [29]	Prospective cohort study, 1995–2003	100 121 135 109 120	⁕	0.275 (nitrite plus nitrate) 0.725 1.225 1.925 3.575	1 (Reference) 1.15 (0.88–1.5) 1.24 (0.95–1.61) 0.97 (0.74–1.28) 1.04 (0.79–1.36)	Glioma	Sex, age, race, energy intake, education, height, and history of cancer at baseline	8
		101 129 106 118 131	⁕	1.13 1.43 1.63 1.85 2.25	1 (Reference) 1.25 (0.96–1.63) 1.03 (0.79–1.36) 1.16 (0.89–1.52) 1.32 (1.01–1.71)			
Mary H. Ward et al., 2005, USA [75]	Case-control study, 1983–1994	38 27 23 33	67 74 71 73	0–0.7 0.7–0.94 0.94–1.19 1.19–1.44	1 (Reference) 0.8 (0.4–1.7) 1.0 (0.4–2.3) 1.2 (0.5–3.2)	Glioma	Age, gender, respondent type, education, ever live/work on a farm, education, beta-carotene, fiber, and calories	6
Dominique S Michaud et al., 2009, USA [32]	3 prospective cohort studies, 1976–2005	55 65 71 69 75	Sub-cohort (PY) =812,763 812,974 844,064 810,417 820,895	⁑ 1.4 1.6 1.8 2.03	1 (Reference) 1.11 (0.72–1.71) 1.2 (0.84–1.71) 1.14 (0.73–1.78) 1.26 (0.89–1.79)	Glioma	Sex, age, race, energy intake, education, height, and history of cancer at baseline	8
Mary H. Ward et al., 2006, USA [56]	Case-control study, 1998- 2000	82 108 110 166	98 98 98 97	0–0.71 0.71–0.909 0.91–1.209 1.21–1.509	1 (Reference) 1.5 (1–2.3) 1.7 (1.1–2.7) 3.1 (1.7–5.5)	Non-Hodgkin’s lymphoma	Age, education, sex, study center, race, dietary vitamin C, and total energy	
Briseis Aschebrook-Kilfoya et al., 2012, USA [58]	Case-control study, 1996–2008	⁕	⁕	0–0.8 0.8–1.1 1.1–1.4 1.4–1.7	1 (Reference) 1.2(0.8–1.6) 0.8 (0.6–1.3) 1 (0.6–1.6)	Non-Hodgkin’s lymphoma	Calories, age, family history, and vitamin C	
Briseis Aschebrook-Kilfoya et al., 2013, USA [14]	Case-control study, 1999–2002	82 90 68 95	114 115 116 114	0.9 1.2 1.5 1.7	1 (Reference) 1.2 (0.8–1.8) 0.8 (0.5–1.3) 1.3 (0.8–1.9)	Non-Hodgkin’s lymphoma	Sex, age, body mass index, education, family history of cancer, vitamin C, and daily caloric intake	
Brian C.-H. Chiu et al., 2008, USA [60]	Case-control study, 1983–1986	14 15 31	357 358 360	0–1 1–1 1–2	1 (Reference) 1.1 (0.5–2.4) 2.8 (1.3–6.1)	Non-Hodgkin’s lymphoma	Age, sex, type of respondent, family history of cancer, and body mass index	8
		39 25 23	357 358 360	0–1 1–1 1–2	1 (Reference) 0.7 (0.4–1.1) 0.6 (0.3–1.2)			
Briseis Aschebrook-Kilfoya et al., 2010, USA [25]	Case-control study, 1995–2001	248 345	349 355	Low High	1 (Reference) 1.37 (1.04–1.79)	Non-Hodgkin’s lymphoma	Age, family history of cancer, calories, vitamin C intake, vitamin E intake, and protein intake	
Arbor J.L. Quist et al., 2018, USA [61]	Cohort study, 1986–2011	88 67 70 68 15	*n* = 8501 8505 8753 6761 1722	0–0.86 0.86–1.11 1.12–1.43 1.44–2.05 2.05–2.66	1 (Reference) 0.85 (0.59–1.22) 0.94 (0.62–1.42) 1.3 (0.79–2.14) 1.28 (0.59–2.79)	Pancreas	Age, smoking category, calories, and mutually adjusted for either natural log-transported nitrate or nitrite	
Angela Coss et al., 2004, USA [62]	Case-control study, 1960–1987	15 22 40 64	233 307 333 374	0–0.75 0.75–0.98 0.99–1.3 1.3–1.61	1 (Reference) 1 (0.52–2) 1.5 (0.81–2.9) 1.5 (0.79–3)	Pancreas	Age, cigarette use, and caloric intake	
		9 22 60 50	264 282 359 342	0–0.22 0.22–0.31 0.32–0.53 0.53–0.74	1 (Reference) 2.1 (0.95–4.8) 3.8 (1.8–8) 2.3 (1.1–5.1)			
		18 32 32 40	144 146 168 181	0–0.56 0.56–0.71 0.72–0.93 0.93–1.13	1 (Reference) 1.8 (0.94–3.4) 1.4 (0.72–2.6) 1.3 (0.65–2.5)			
		13 32 26 51	148 164 147 180	0–0.13 0.13–0.18 0.19–0.26 0.26–0.33	1 (Reference) 2.4 (1.2–4.7) 1.9 (0.94–4) 3.2 (1.6–6.4)			
* Jiali Zheng et al., 2019, USA [63]	Case–control study, 2002–2009	291 226 225 215	235 234 235 234	0.025–1.475 1.375–2.1 2.075–2.925 2.9–9.65	1 (Reference) 0.8 (0.62–1.03) 0.77 (0.6–1) 0.75 (0.59–0.91)	Pancreas	Age and energy intake	
Briseis Aschebrook-Kilfoy et al., 2011, USA [64]	Prospective cohort study, 1995- 2006	361 361 331 348 321	⁕	0.8 1.0 1.2 1.2 1.6	1(Reference) 0.99 (0.86–1.16) 0.92 (0.79–1.08) 0.97 (0.83–1.14) 0.92 (0.78–1.08)	Pancreas	Age, race, total energy intake, smoking status, family history of cancer, family history of diabetes, body mass index, and intakes of saturated fat, folate, and vitamin C	
Rena R. Jones et al., 2016, USA [8]	Cohort study, 1986–2010	63 66 73 56	*n* = 8450 8514 8487 8513	0–0.86 0.86–1.12 1.13–1.43 1.44–1.74	1 (Reference) 1.15 (0.78–1.7) 1.38 (0.89–2.16) 1.15 (0.65–2.03)	Bladder	Age, smoking status, pack-years of smoking, in-transformed total energy intake, and total in-transformed dietary nitrate from all sources	
Kathryn Hughes Barry et al., 2020, New England [76]	Case–control study, 1994–1996, 2001–2004	222 212 202 217 184	243 245 244 248 245	0–0.48 0.48–0.56 0.56–0.63 0.63–0.72 >0.72	1 (Reference) 1.0 (0.77–1.4) 1.0 (0.74–1.3) 1.1 (0.80–1.4) 0.97 (0.71–1.3)	Bladder	Adjusted for age, gender, smoking status, high-risk occupation, race, ethnicity, state, dietary vitamin C intake (per 1000 kcal—continuous), dietary vitamin B12 (per 1000 kcal—continuous), total energy intake (kcal—continuous), and total water intake (L/d—continuous); models for nitrate/nitrite from processed meat were additionally adjusted for total meat intake (per 1000 kcal—continuous)	
Mary H. Ward et al., 2003, USA [35]	Case-control study, 1986–1989	⁕	⁕	0–0.81 0.81–1.06 1.06–1.39 1.39–1.72	1 (Reference) 1.1 (0.9–1.4) 1.2 (0.9–1.5) 1.2 (0.9–1.6)	Bladder	Age, education, and cigarette smoking, years chlorinated surface water, and study period	
		⁕	⁕	0–0.58 0.58–0.75 0.75–0.98 0.98–1.21	1 (Reference) 1 (0.6–1.5) 0.8 (0.5–1.3) 1 (0.7–1.6)			
* Leah M. Ferrucci et al., 2010, USA [5]	Cohort study, 1995– 2003	176 181 164 161 172	⁕	1.15 1.425 1.625 1.85 2.275	1 (Reference) 1.17 (0.9–1.45) 1.1 (0.89–1.37) 1.14 (0.91–1.44) 1.28 (1.02–1.61)	Bladder	Age, gender, smoking, intakes of fruit, vegetables, beverages, and total energy	
		109 147 173 191 234	⁕	0.025 0.075 0.15 0.25 0.475	1 (Reference) 1.15 (0.9–1.46) 1.08 (0.85–1.37) 1.39 (1.11–1.74) 1.07 (0.85–1.36)			
* Chelsea E. Catsburg et al., 2014, USA [38]	Case-control study, 1987– 1996	400 287 302 314 344	314 316 313 315 315	0–0.234 0.235–0.311 0.312–0.4 0.401–0.532 0.532–0.664	1 (Reference) 0.75 (0.59–0.94) 0.81 (0.63–1.03) 0.82 (0.64–1.07) 0.89 (0.66–1.2)	Bladder	Smoking duration, smoking intensity, and smoking status	8
Mary H. Ward et al., 2007, USA [27]	Case-control study, 1986–1989	92 74 78 89	471 472 471 472	0–0.7 0.7–0.93 0.94–1.25 1.26–1.57	1 (Reference) 0.82 (0.58–1.17) 0.84 (0.57–1.22) 0.82 (0.5–1.33)	Kidney	Age, gender, sodium, total fat, and total calories	
		64 90 88 91	471 472 471 472	0–0.18 0.18–0.28 0.29–0.47 0.48–0.66	1 (Reference) 1.37 (0.95–1.95) 1.24 (0.85–1.83) 1 (0.63–1.59)			
Rena R. Jones et al., 2017, USA [67]	Cohort study, 1986–2010	57 68 69 49 13	*n* = 8450 8514 8487 1704 6809	0–0.86 0.86–1.12 1.13–1.43 1.44–2.06 2.06–2.68	1 (Reference) 1.3 (0.87–1.9) 1.4 (0.89–2.3) 1.4 (0.77–2.5) 1.6 (0.7–3.8)	Kidney	Age, smoking status, pack-years of smoking, in-transformed total energy intake, body mass index, and total in-transformed total dietary nitrate or nitrite	
Mary H. Ward et al., 2008, USA [68]	Case-control study, 1988–1994	23 28 17 30	94 102 101 100	0–0.36 0.36–0.52 0.52–0.67 0.67–0.82	1 (Reference) 1.1 (0.5–2.3) 0.6 (0.2–1.3) 1 (0.4–2.4)	Esophagus	Year of birth, gender, body mass index, smoking, alcohol, total calories, vitamin A, folate, riboflavin, zinc, protein, and carbohydrate	
		19 31 25 29	99 99 99 100	0–3.8 (nitrite plus nitrate) 3.8–5.7 5.7–8.3 8.3–10.9	1 (Reference) 0.7 (0.3–1.6) 1.7 (0.7–4.1) 2.2 (0.9–5.7)			
Andra´s P Keszei et al., 2013, The Netherlands [69]	Cohort study, 1986–2002	17 19 23	Sub-cohort (PY) 8665 8895 8890	0.03 0.12 0.28	1 (Reference) 1.18 (0.61–2.3) 1.49 (0.78–2.87)	Esophagus	Age	
		42 38 34	8665 8895 8890	0.03 0.12 0.28	1(Reference) 0.9 (0.57–1.43) 0.81 (0.5–1.31)			
		16 18 14	10,009 10,016 9752	0.02 0.08 0.2	1 (Reference) 1.17 (0.59–2.32) 0.96 (0.46–2)			
		12 12 7	10,009 10,016 9752	0.02 0.08 0.2	1 (Reference) 1.05 (0.47–2.36) 0.64 (0.25–1.64)			
* Amanda J. Cross et al., 2011, USA [24]	Cohort study, 1995–2006	20 30 19 28 31	⁕	0.0303 0.0865 0.1535 0.2573 0.498	1 (Reference) 1.36 (0.76–2.43) 0.82 (0.43–1.57) 1.15 (0.63–2.11) 1.21 (0.67–2.2)	Esophagus	Age, sex, BMI, education, ethnicity, tobacco smoking, alcohol drinking, usual physical activity at work, vigorous physical activity, daily intake of fruit, vegetables, saturated fat, and calories	
		50 60 66 81 120	⁕	0.0303 0.0865 0.1535 0.2573 0.498	1 (Reference) 0.89 (0.61–1.3) 0.82 (0.56–1.2) 0.88 (0.61–1.27) 1.19 (0.84–1.68)			
Lawrence S. Engel et al., 2003, USA [77]	Case–control study, 1993–1995	⁕	⁕	1.8–5.55 5.65–7.2 7.3–9.5 9.6–35.2	1 (Reference) 1.5 (1–2.4) 1.8 (1.1–3) 2.5(1.4–4.3)	Stomach	Geographic center, age, sex, race, income, respondent type, energy intake, and the other factors included in the table	
A. J. M. van Loon et al., 1998, The Netherlands [70]	Cohort study, 1986–1992	47 51 58 46 80	Sub-cohort (PY) 3873 3706 3829 3844 3760	0.01 0.04 0.09 0.16 0.35	1 (Reference) 1.15 (0.76–1.74) 1.21 (0.81–1.83) 0.87 (0.57–1.33) 1.49 (1.01–2.2)	Stomach	Age and sex	
Raúl U. Hernández-Ramírez et al., 2009, Mexico [71]	Case–control study, 2004–2005	⁕	⁕	0–1 1–1.2 1.2–1.4	1 (Reference) 1.07 (0.69–1.65) 1.52 (0.99–2.34)	Stomach	Energy, age, gender, *H. pylori* CagA status, schooling, and consumptions of salt, chili, and alcohol	
Andra´s P Keszei et al., 2013, The Netherlands [69]	Cohort study, 1986–2002	47 39 53	Sub-cohort (PY) 8665 8895 8890	0.03 0.12 0.28	1 (Reference) 0.83 (0.53–1.29) 1.14 (0.75–1.72)	Stomach	Age	
		98 109 122	8665 8895 8890	0.03 0.12 0.28	1 (Reference) 1.17 (0.87–1.58) 1.36 (1.01–1.82)			
		9 9 6	10,009 10,016 9752	0.02 0.08 0.2	1 (Reference) 1.05 (0.41–2.67) 0.73 (0.26–2.07)			
		56 50 54	10,009 10,016 9752	0.02 0.08 0.2	1 (Reference) 0.94 (0.63–1.39) 1.06 (0.71–1.57)			
Carlo La Vecchia et al., 1994, Italy [72]	Case-control study, 1985–1992	⁕	⁕	1.91 2.41 2.94 3.64	1 (Reference) 0.96 (0.69–1.32) 0.97 (0.7–1.35) 1.02 (0.73–1.43)	Stomach	Age, sex, education, family history of gastric cancer, body mass index, total energy intake, plus all the above variables	5
* Amanda J. Cross et al., 2011, USA [24]	Cohort study, 1995–2006	44 40 55 61 55	⁕	0.0303 0.0865 0.1535 0.2573 0.498	1 (Reference) 0.72 (0.47–1.11) 0.88 (0.58–1.32) 0.87 (0.58–1.31) 0.71 (0.47–1.08)	Stomach	Age, sex, BMI, education, ethnicity, tobacco smoking, alcohol drinking, usual physical activity at work, vigorous physical activity, daily intake of fruits, vegetables, saturated fat, and calories	
		54 44 48 67 64	⁕	0.0303 0.0865 0.1535 0.2573 0.498	1 (Reference) 0.77 (0.51–1.15) 0.79 (0.53–1.18) 1.04 (0.71–1.52) 0.93 (0.63–1.37)			
Mary H. Ward, et al., 2008, USA [68]	Case-control study, 1988–1994	23 22 29 30	94 102 101 100	0–0.36 0.36–0.52 0.52–0.67 0.67–0.83	1 (Reference) 1.1 (0.4–2.7) 0.8 (0.3–2.2) 1.1 (0.3–3.4)	Stomach	Year of birth, gender, education, smoking, alcohol, total calories, vitamin C, fiber, and carbohydrate	
		19 31 25 29	99 99 99 100	0–3.8 3.8–5.7 5.7–8.3 8.3–10.9	1 (Reference) 1.6 (0.8–3.2) 1.8 (0.8–3.8) 1.6 (0.7–3.7)			
Curt T. DellaValle et al., 2014, China [44]	Prospective cohort study, 1996–2007	72 81 75 80 75	⁕	0.56 0.74 0.87 1.01 1.23	1 (Reference) 1.27 (0.92–1.76) 1.23 (0.88–1.73) 1.34 (0.94–1.9) 1.26 (0.85–1.86)	Colon	Age, energy intake, education, physical activity, dietary vitamin C intake, carotene, and folate	
Rena R. Jones et al., 2019, USA [41]	Cohort study, 1986– 2010	345 342 320 317	*n* = 8588 8655 8974 8491	0–0.57 0.58–0.65 0.66–0.74 0.74–0.82	1 (Reference) 0.93 (0.8–1.08) 0.83 (0.71–0.97) 0.87 (0.74–1.02)	Colon	Age, heme iron, red meat, and total dietary nitrate or nitrite	
* Amanda J. Cross et al., 2010, USA [29]	Prospective cohort study, 1994–2003	344 359 397 441 454	⁕	0.0298 0.0843 0.1493 0.2498 0.4853	1 (Reference) 0.96 (0.83–1.12) 1.01 (0.88–1.18) 1.09 (0.94–1.26) 1.09 (0.94–1.26)	Colon	Gender, education, BMI, smoking, and intake of total energy, fiber, and dietary calcium	
* L. M. Ferrucci et al., 2012, USA [73]	Multi-center, randomized controlled trial 1993–2001	150 165 203 254	⁕	0.15 (nitrite plus nitrate) 0.425 0.9 2.1	1 (Reference) 0.98 (0.77–1.23) 1.07 (0.84–1.35) 1.16 (0.9–1.5)	Colon	Age, study center, gender, ethnicity, education, family history of colorectal cancer, BMI, NSAIDs use, physical activity, smoking status, alcohol intake, dietary calcium, supplemental calcium, dietary fiber, and total energy intake	
Anneclaire J. De Roos et al., 2003, USA [28]	Case-Control study, 1986–1990	(*n*(%)) 90 (32) 73 (26) 48 (17) 69 (25)	(*n*(%)) 311 (32) 251 (26) 220 (22) 200 (20)	0–0.704 0.705–0.93 0.94–1.25 1.26–1.57	1 (Reference) 1.1 (0.8–1.6) 0.9 (0.6–1.3) 1.5 (1–2.1)	Colon	Age, sex, and chlorinated surface water	
Yun Zhu et al. 2014 [74]	Case-control study, 1997–2006	131 145 126 120 139	536 496 520 474 455	0.65 0.89 1.12 1.4 1.92	1 (Reference) 1.15 (0.86–1.54) 0.91 (0.66–1.26) 0.81 (0.56–1.18) 0.95 (0.63–1.43)	Colon	Age, sex, energy intake, BMI, cigarette smoking status, education attainment, reported colon screening procedures, NSAID use, multivitamin supplements use, folate supplement use, vegetable intakes, and province of residence	
		107 112 101 132 134	536 496 520 474 455	0.65 0.89 1.12 1.4 1.92	1 (Reference) 0.97 (0.7–1.34) 0.93 (0.65–1.32) 1.21 (0.82–1.78) 1.32 (0.85–2.04)			
Curt T. Della Valle et al., 2014, China [44]	Prospective cohort study, 1996–2007	57 45 48 42 44	⁕	0.56 0.74 0.87 1.01 1.23	1 (Reference) 0.87 (0.58–1.29) 0.94 (0.63–1.42) 0.81 (0.52–1.25) 0.8 (0.49–1.29)	Rectum	Age, energy intake, education, physical activity, dietary vitamin C intake, carotene, and folate	
Rena R. Jones et al., 2019, USA [41]	Cohort study, 1986– 2010	93 74 91 67	*n* = 8588 8655 8974 8491	0–0.57 0.58–0.65 0.66–0.74 0.74–0.82	1 (Reference) 0.75 (0.55–1.02) 0.88 (0.65–1.18) 0.68 (0.49–0.94)	Rectum	Age and total dietary nitrate or nitrite	
* Amanda J. Cross et al., 2010, USA [29]	Prospective cohort study, 1994–2003	113 129 157 162 163	⁕	0.0298 0.0843 0.1493 0.2498 0.4853	1 (Reference) 1.07 (0.83–1.38) 1.23 (0.96–1.58) 1.21 (0.94–1.55) 1.16 (0.9–1.5)	Rectum	Gender, education, BMI, smoking, and intake of total energy, fiber, and dietary calcium	
* L. M. Ferrucci et al., 2012, USA [73]	Multi-center, randomized controlled trial 1993–2001	44 64 75 80	⁕	0.15 (nitrite plus nitrate) 0.425 0.9 2.1	1 (Reference) 1.31 (0.88–1.95) 1.38 (0.92–2.07) 1.27 (0.8–1.99)	Rectum	Age, study center, gender, ethnicity, education, family history of colorectal cancer, BMI, NSAIDs use, physical activity, smoking status, alcohol intake, dietary calcium, supplemental calcium, dietary fiber, and total energy intake	
Anneclaire J. De Roos et al., 2003, USA [28]	Case-control study, 1986–1990	(*n*(%)) 74 (30) 62 (25) 43 (17) 70 (28)	(*n*(%)) 311 (32) 251 (26) 220 (22) 200 (20)	0–0.705 0.705–0.93 0.94–1.25 1.26–1.57	1 (Reference) 1.1 (0.7–1.6) 0.9 (0.6–1.4) 1.7 (1.1–2.5)	Rectum	Age, sex, and chlorinated surface water	
Yun Zhu et al. 2014 [74]	Case-control study, 1997–2006	95 120 124 145 141	536 496 520 474 455	0.65 0.89 1.12 1.4 1.92	1 (Reference) 1.26 (0.91–1.73) 1.2 (0.84–1.71) 1.51 (1.02–2.22) 1.45 (0.94–2.24)	Rectum	Age, sex, energy intake, BMI, cigarette smoking status, education attainment, reported colon screening procedures, NSAID use, multivitamin supplements use, folate supplement use, vegetable intakes, and province of residence	

* Original exposure categories of nitrite from studies converted to mg/day for meta-regression calculation (explained under statistics analysis); ⁕ missing cases/controls/person-years in sub-cohort from the studies; ⁑ missing nitrite dosage.

## Data Availability

All data generated or analyzed during this study are included in this published article. The data set used/analyzed are available from the corresponding author on request.

## Supplementary Materials

The following supporting information can be downloaded at: https://www.mdpi.com/article/10.3390/nu14030666/s1, Table S1: Search Strategy of this study; Figure S1: Pooled ORs (95 % CI) of the highest dosage versus lowest category of dosage of nitrate and nitrite consumption from dietary intake for the following cancer type: (1) Reproductive organs, (2) Breast, (3) Thyroid, (4) Glioma, (5) Non-hodgkin, (6) Pancreatic, (7) Bladder, (8) Kidney, (9) Esophageal, (10) Stomach, (11) Colon, and (12) Rectum; Figure S2: Pooled ORs (95 % CI) of all combined higher dosages versus the lowest category of nitrate and nitrite consumption from dietary intake for each type of cancer; Figure S3: Funnel plot of nitrates and nitrites and for each type of cancer risk for publication bias.
