# Supplementary material for: Association between Dietary Nitrate, Nitrite Intake, and Site-Specific Cancer Risk: A Systematic Review and Meta-Analysis
Source: Nutrients. 2022 Feb 4;14(3):666. doi: 10.3390/nu14030666 (PMC8838348; doi:10.3390/nu14030666)
Supplement: Supplementary file 1 [file nutrients-14-00666-s001.zip › nutrients-1489942-supplementary.pptx]

## Slide 1
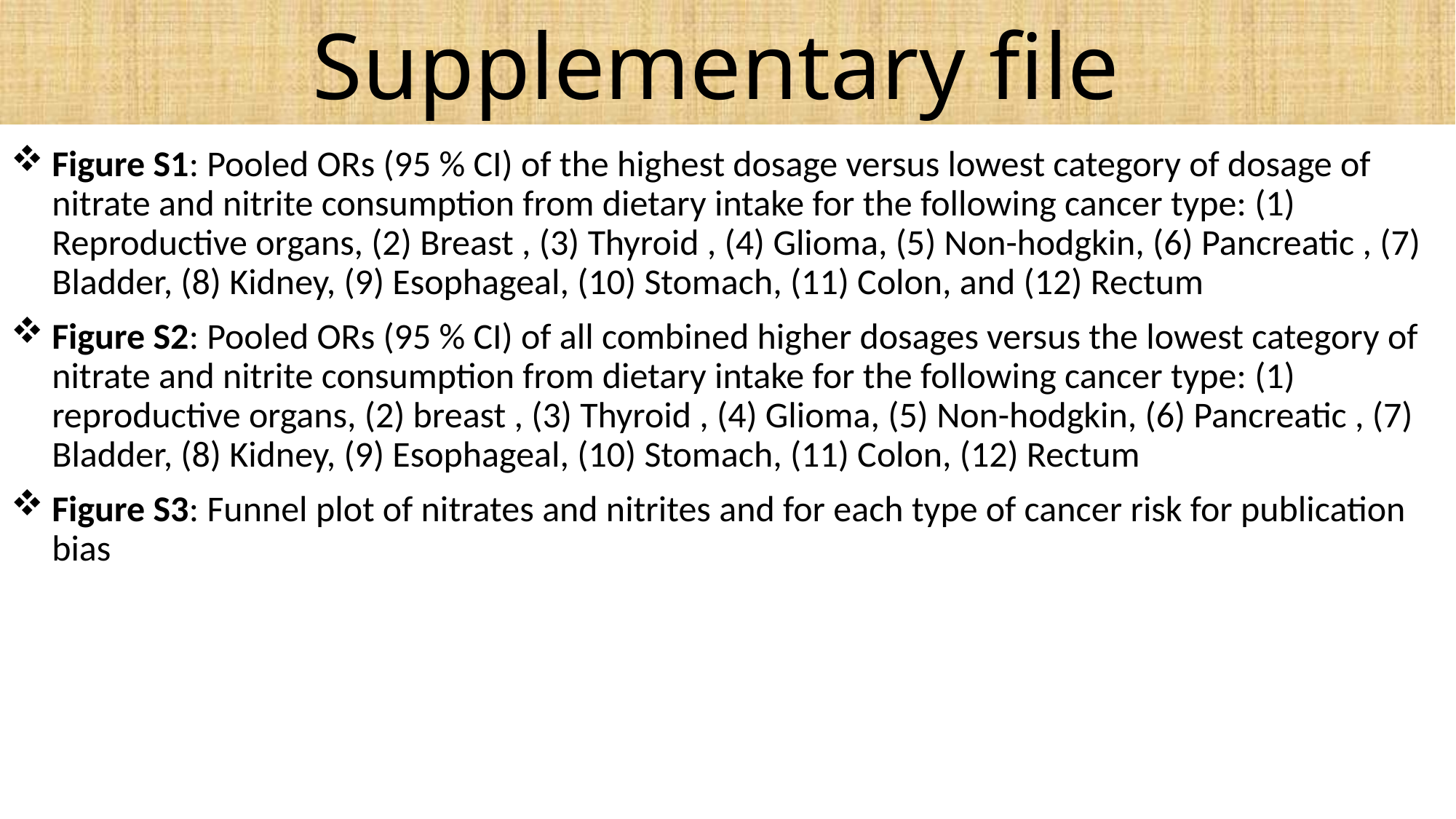

# Supplementary file
Figure S1: Pooled ORs (95 % CI) of the highest dosage versus lowest category of dosage of nitrate and nitrite consumption from dietary intake for the following cancer type: (1) Reproductive organs, (2) Breast , (3) Thyroid , (4) Glioma, (5) Non-hodgkin, (6) Pancreatic , (7) Bladder, (8) Kidney, (9) Esophageal, (10) Stomach, (11) Colon, and (12) Rectum
Figure S2: Pooled ORs (95 % CI) of all combined higher dosages versus the lowest category of nitrate and nitrite consumption from dietary intake for the following cancer type: (1) reproductive organs, (2) breast , (3) Thyroid , (4) Glioma, (5) Non-hodgkin, (6) Pancreatic , (7) Bladder, (8) Kidney, (9) Esophageal, (10) Stomach, (11) Colon, (12) Rectum
Figure S3: Funnel plot of nitrates and nitrites and for each type of cancer risk for publication bias

## Slide 2
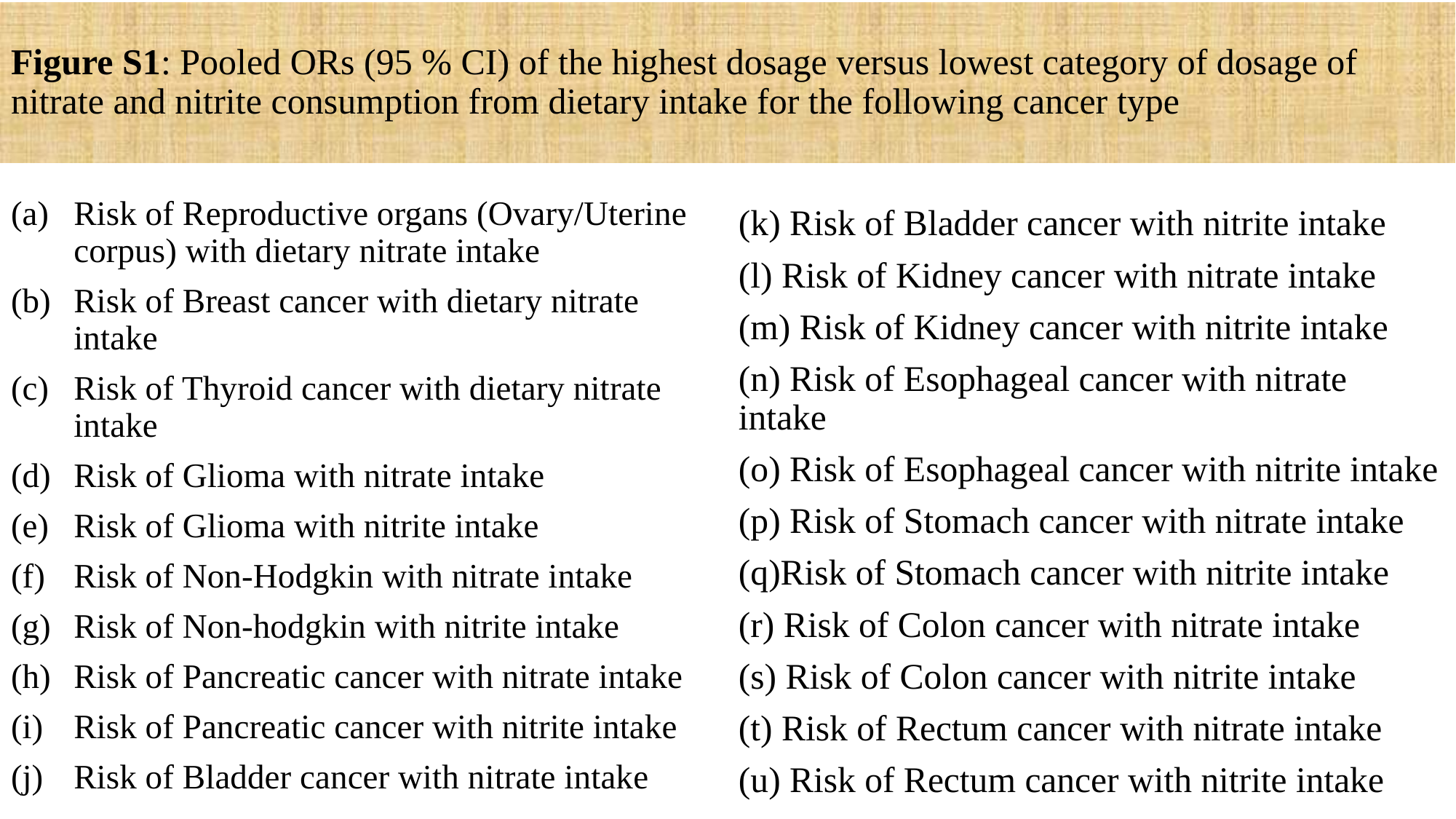

# Figure S1: Pooled ORs (95 % CI) of the highest dosage versus lowest category of dosage of nitrate and nitrite consumption from dietary intake for the following cancer type
Risk of Reproductive organs (Ovary/Uterine corpus) with dietary nitrate intake
Risk of Breast cancer with dietary nitrate intake
Risk of Thyroid cancer with dietary nitrate intake
Risk of Glioma with nitrate intake
Risk of Glioma with nitrite intake
Risk of Non-Hodgkin with nitrate intake
Risk of Non-hodgkin with nitrite intake
Risk of Pancreatic cancer with nitrate intake
Risk of Pancreatic cancer with nitrite intake
Risk of Bladder cancer with nitrate intake
(k) Risk of Bladder cancer with nitrite intake
(l) Risk of Kidney cancer with nitrate intake
(m) Risk of Kidney cancer with nitrite intake
(n) Risk of Esophageal cancer with nitrate intake
(o) Risk of Esophageal cancer with nitrite intake
(p) Risk of Stomach cancer with nitrate intake
(q)Risk of Stomach cancer with nitrite intake
(r) Risk of Colon cancer with nitrate intake
(s) Risk of Colon cancer with nitrite intake
(t) Risk of Rectum cancer with nitrate intake
(u) Risk of Rectum cancer with nitrite intake

## Slide 3
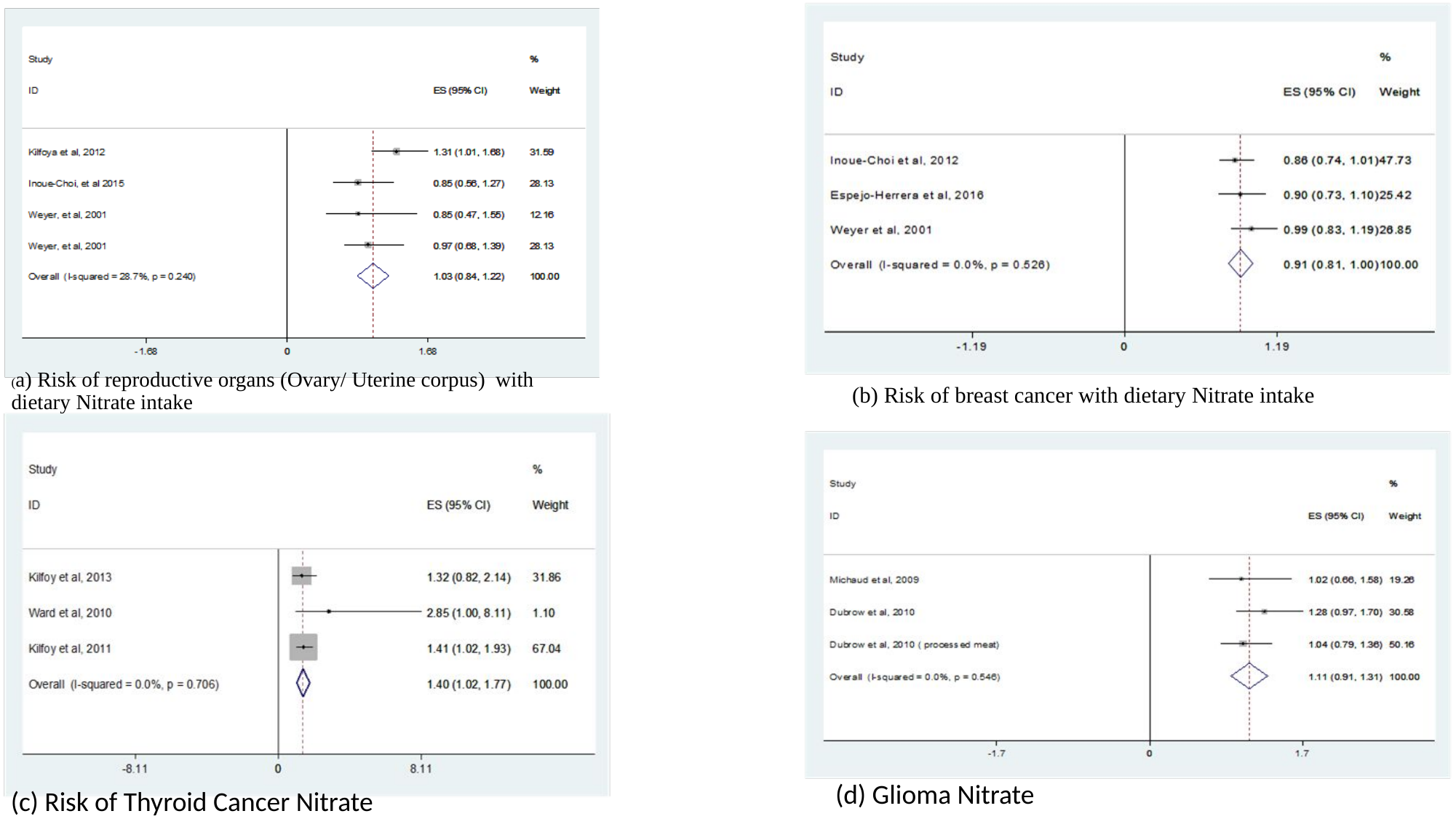

(a) Risk of reproductive organs (Ovary/ Uterine corpus) with dietary Nitrate intake
(b) Risk of breast cancer with dietary Nitrate intake
 (d) Glioma Nitrate
(c) Risk of Thyroid Cancer Nitrate

## Slide 4
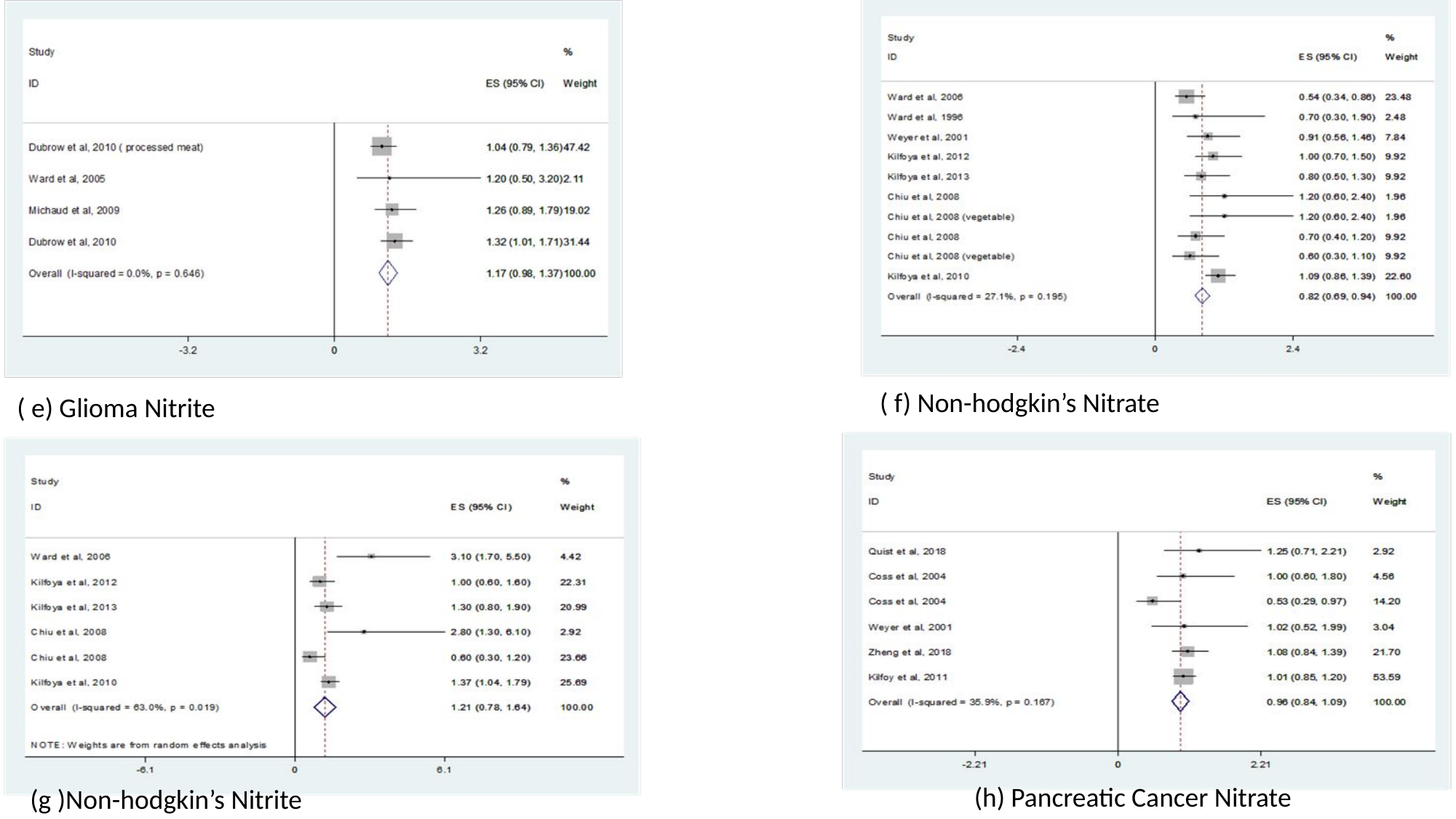

( f) Non-hodgkin’s Nitrate
 ( e) Glioma Nitrite
 (h) Pancreatic Cancer Nitrate
 (g )Non-hodgkin’s Nitrite

## Slide 5
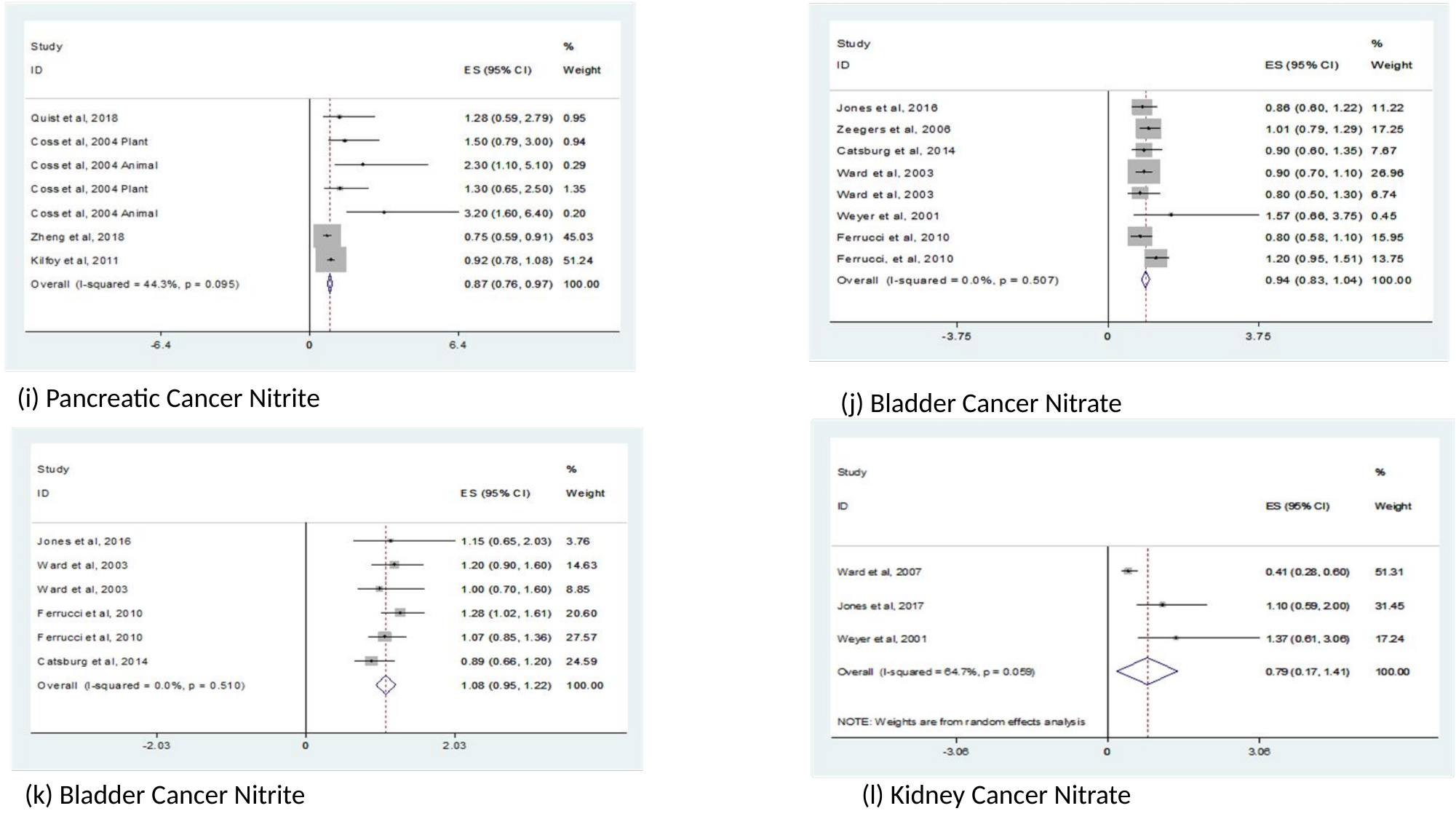

(i) Pancreatic Cancer Nitrite
 (j) Bladder Cancer Nitrate
 (k) Bladder Cancer Nitrite
(l) Kidney Cancer Nitrate

## Slide 6
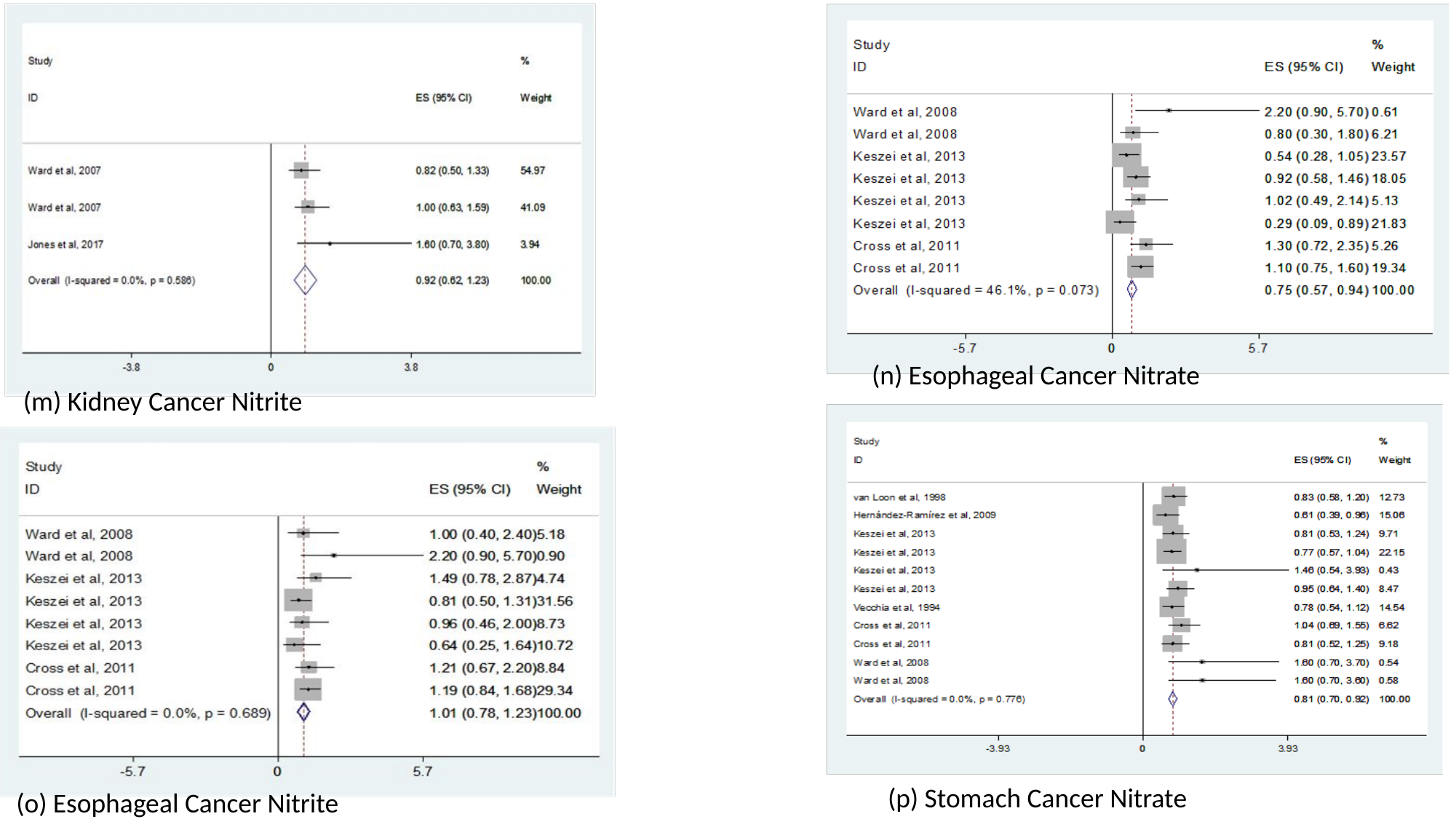

(n) Esophageal Cancer Nitrate
 (m) Kidney Cancer Nitrite
(p) Stomach Cancer Nitrate
 (o) Esophageal Cancer Nitrite

## Slide 7
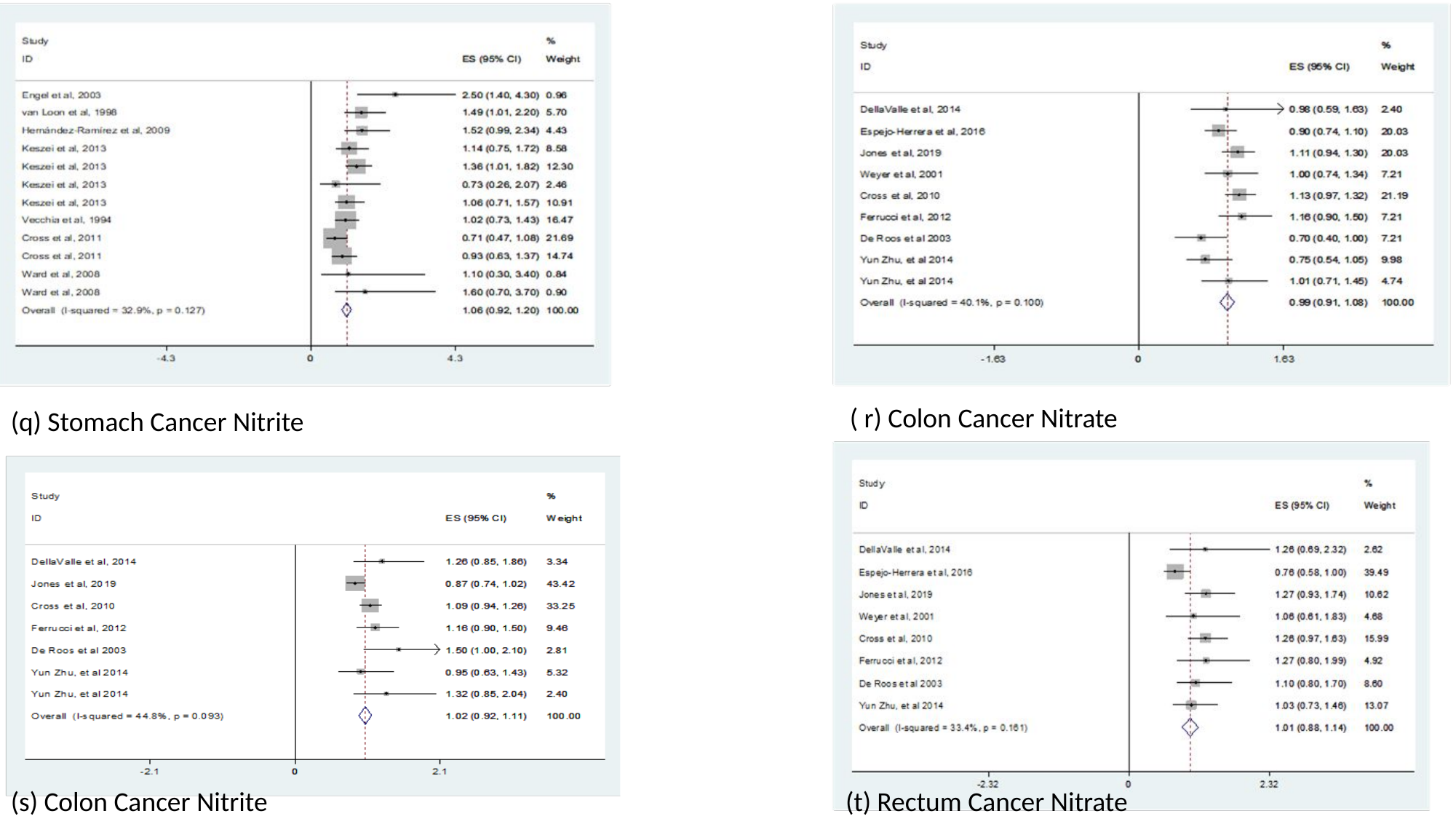

( r) Colon Cancer Nitrate
(q) Stomach Cancer Nitrite
(s) Colon Cancer Nitrite
 (t) Rectum Cancer Nitrate

## Slide 8
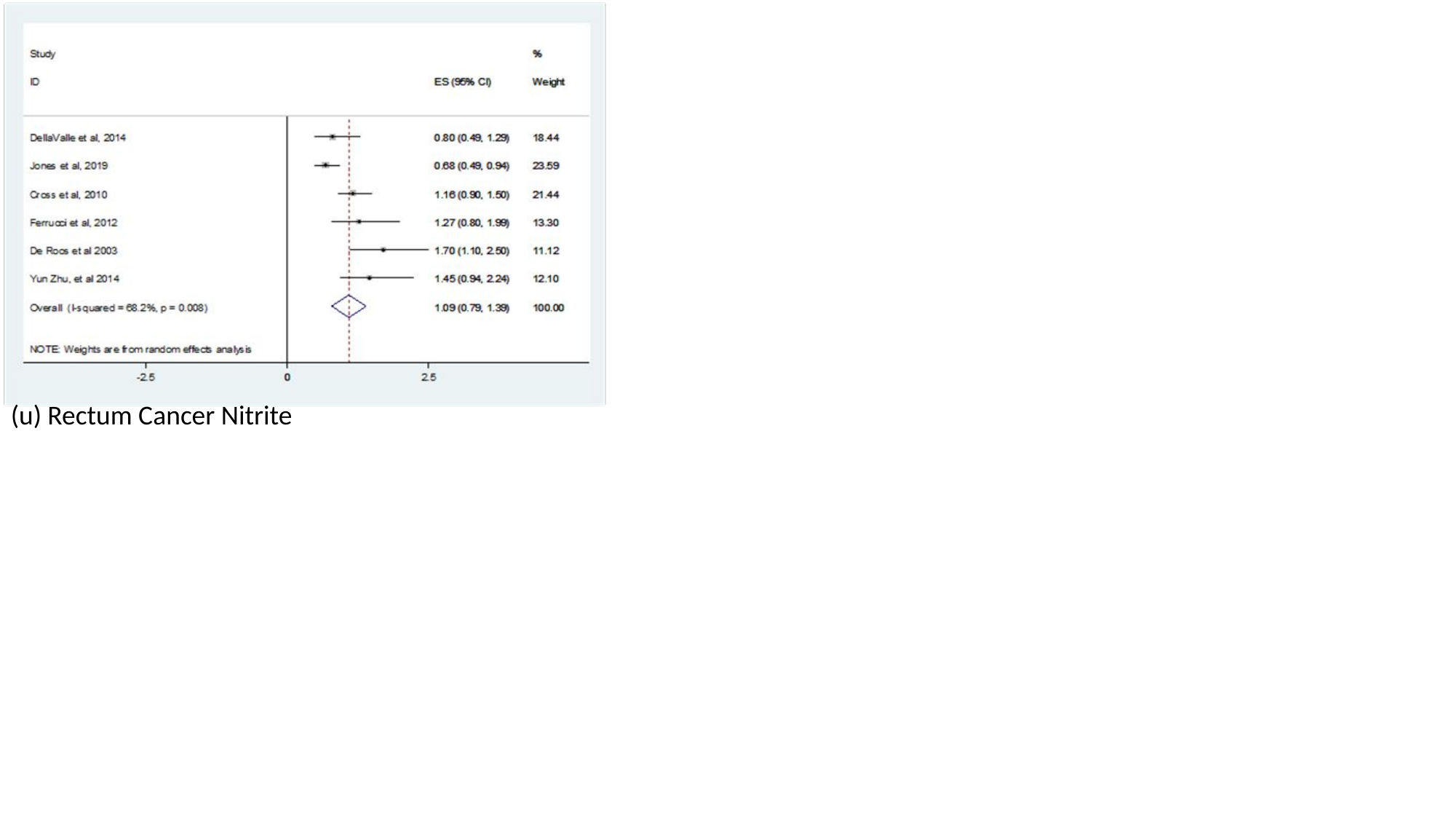

(u) Rectum Cancer Nitrite

## Slide 9
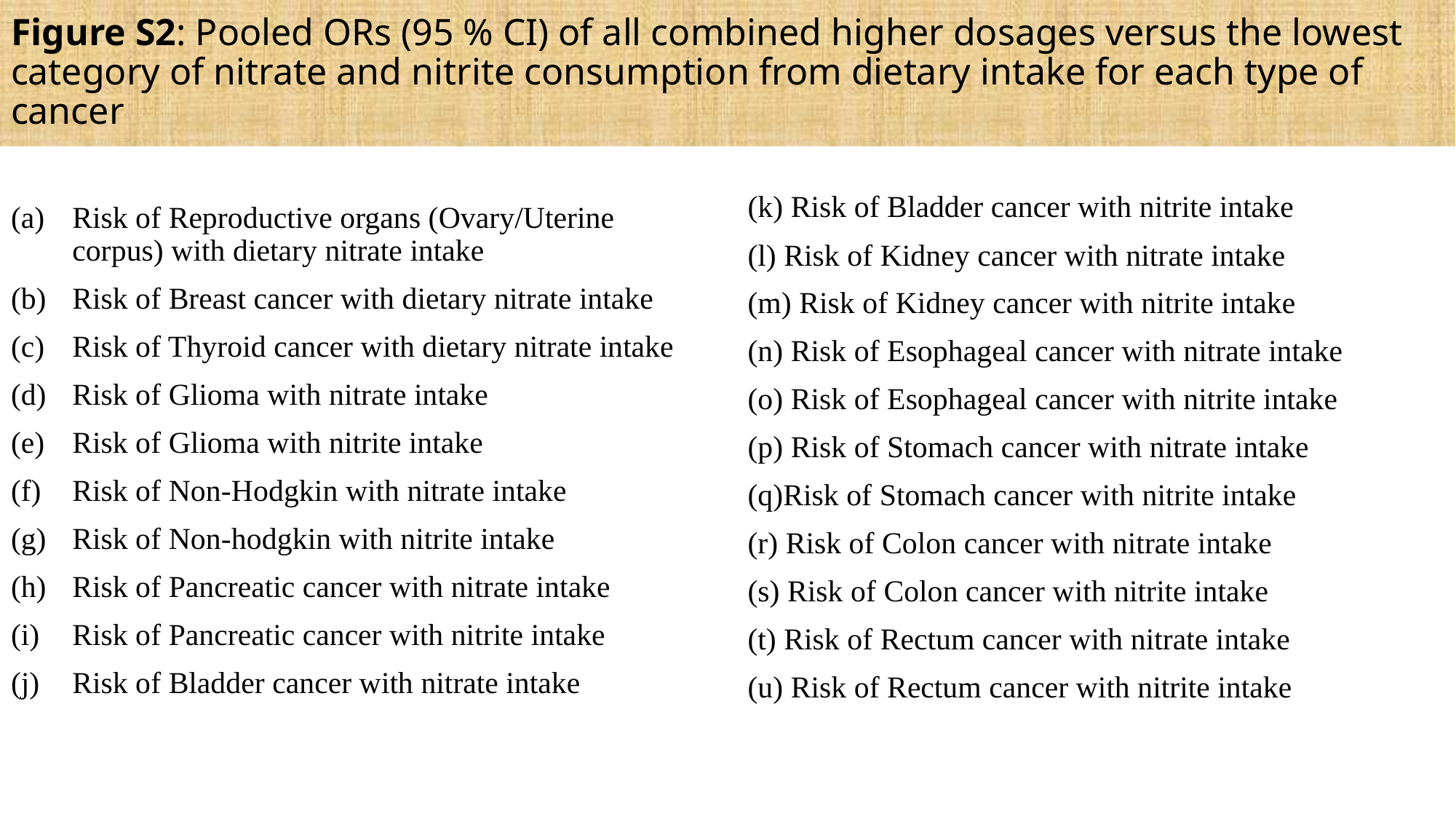

# Figure S2: Pooled ORs (95 % CI) of all combined higher dosages versus the lowest category of nitrate and nitrite consumption from dietary intake for each type of cancer
(k) Risk of Bladder cancer with nitrite intake
(l) Risk of Kidney cancer with nitrate intake
(m) Risk of Kidney cancer with nitrite intake
(n) Risk of Esophageal cancer with nitrate intake
(o) Risk of Esophageal cancer with nitrite intake
(p) Risk of Stomach cancer with nitrate intake
(q)Risk of Stomach cancer with nitrite intake
(r) Risk of Colon cancer with nitrate intake
(s) Risk of Colon cancer with nitrite intake
(t) Risk of Rectum cancer with nitrate intake
(u) Risk of Rectum cancer with nitrite intake
Risk of Reproductive organs (Ovary/Uterine corpus) with dietary nitrate intake
Risk of Breast cancer with dietary nitrate intake
Risk of Thyroid cancer with dietary nitrate intake
Risk of Glioma with nitrate intake
Risk of Glioma with nitrite intake
Risk of Non-Hodgkin with nitrate intake
Risk of Non-hodgkin with nitrite intake
Risk of Pancreatic cancer with nitrate intake
Risk of Pancreatic cancer with nitrite intake
Risk of Bladder cancer with nitrate intake

## Slide 10
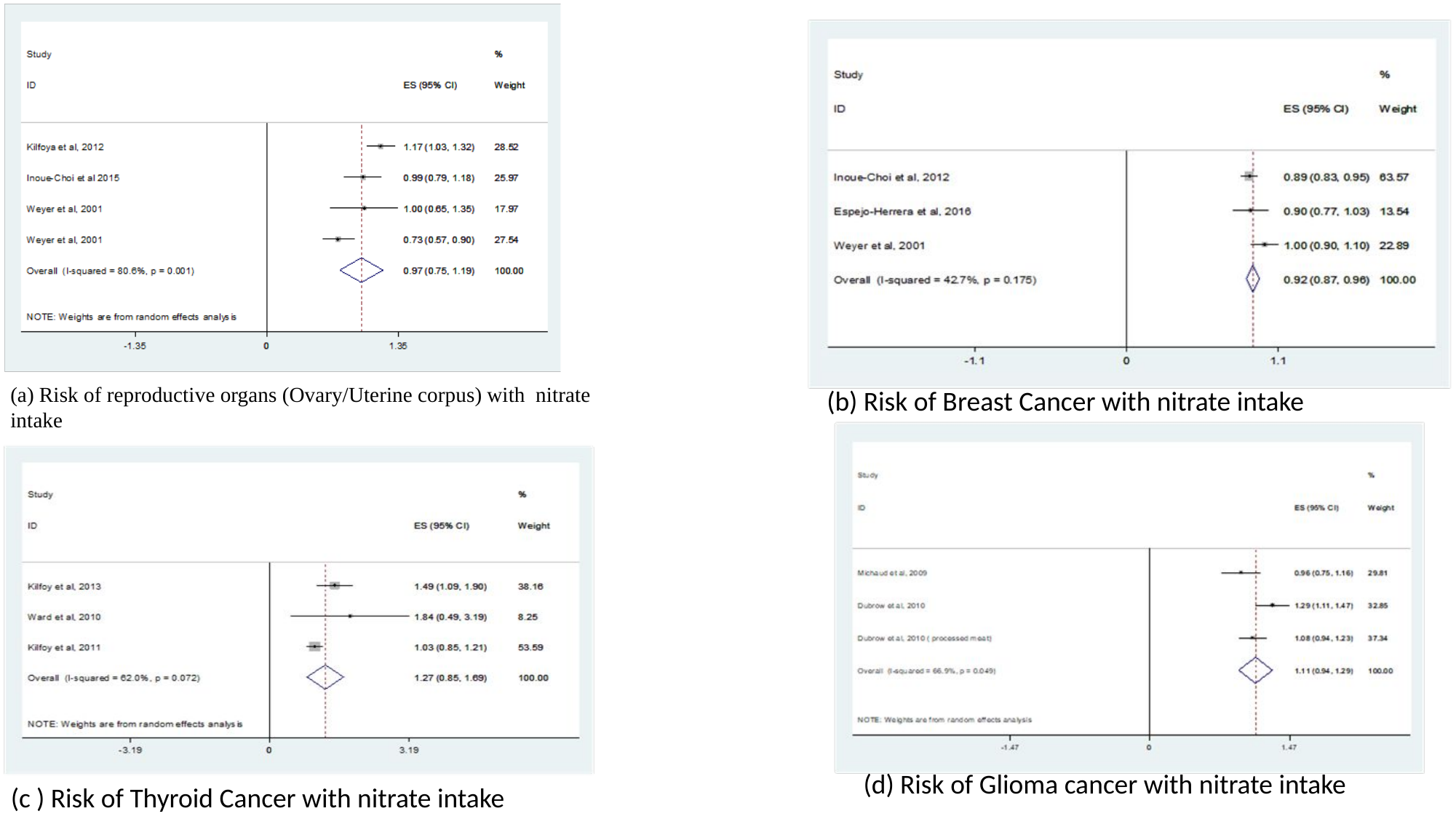

(a) Risk of reproductive organs (Ovary/Uterine corpus) with nitrate intake
(b) Risk of Breast Cancer with nitrate intake
 (d) Risk of Glioma cancer with nitrate intake
(c ) Risk of Thyroid Cancer with nitrate intake

## Slide 11
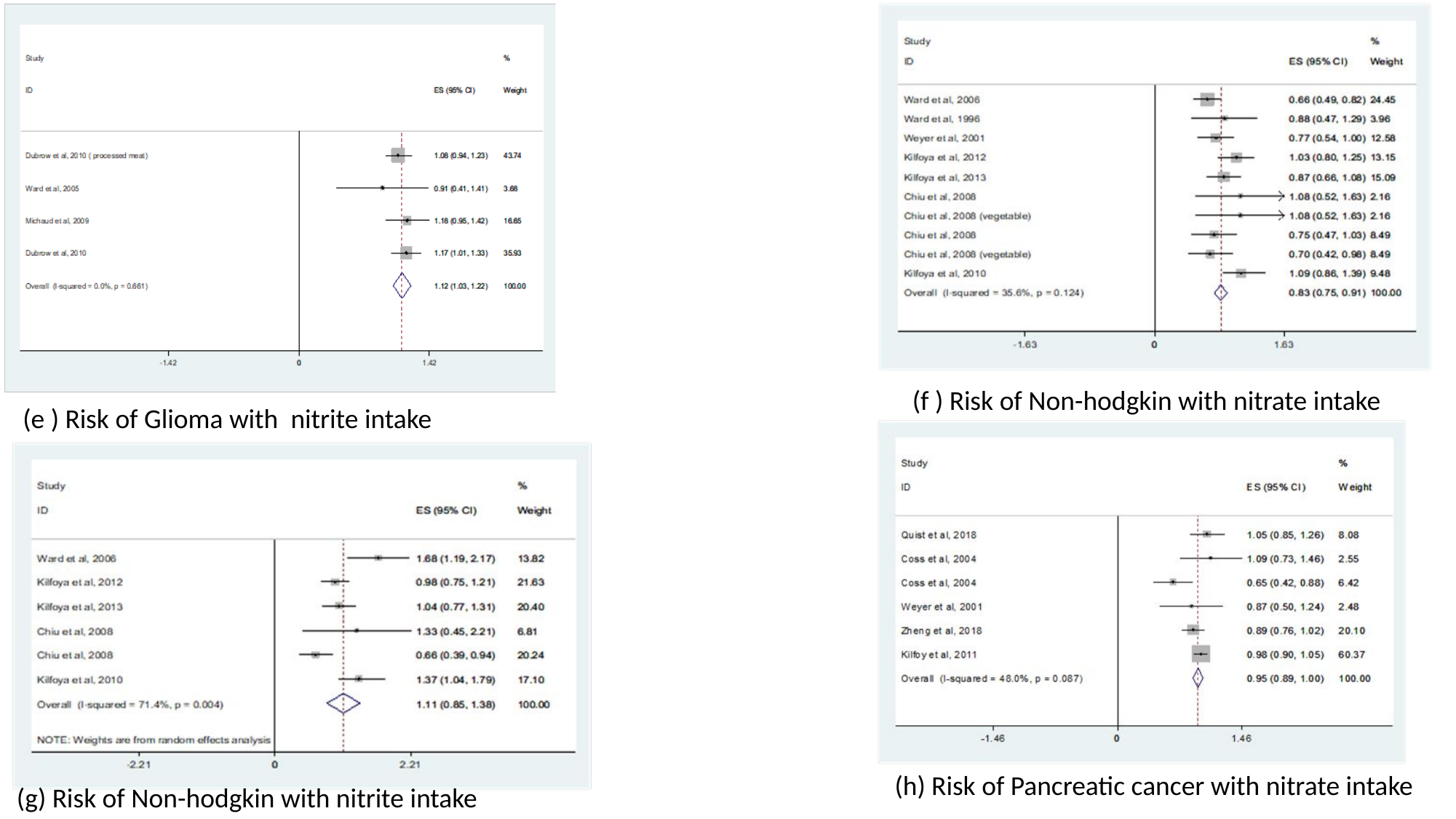

(f ) Risk of Non-hodgkin with nitrate intake
 (e ) Risk of Glioma with nitrite intake
 (h) Risk of Pancreatic cancer with nitrate intake
 (g) Risk of Non-hodgkin with nitrite intake

## Slide 12
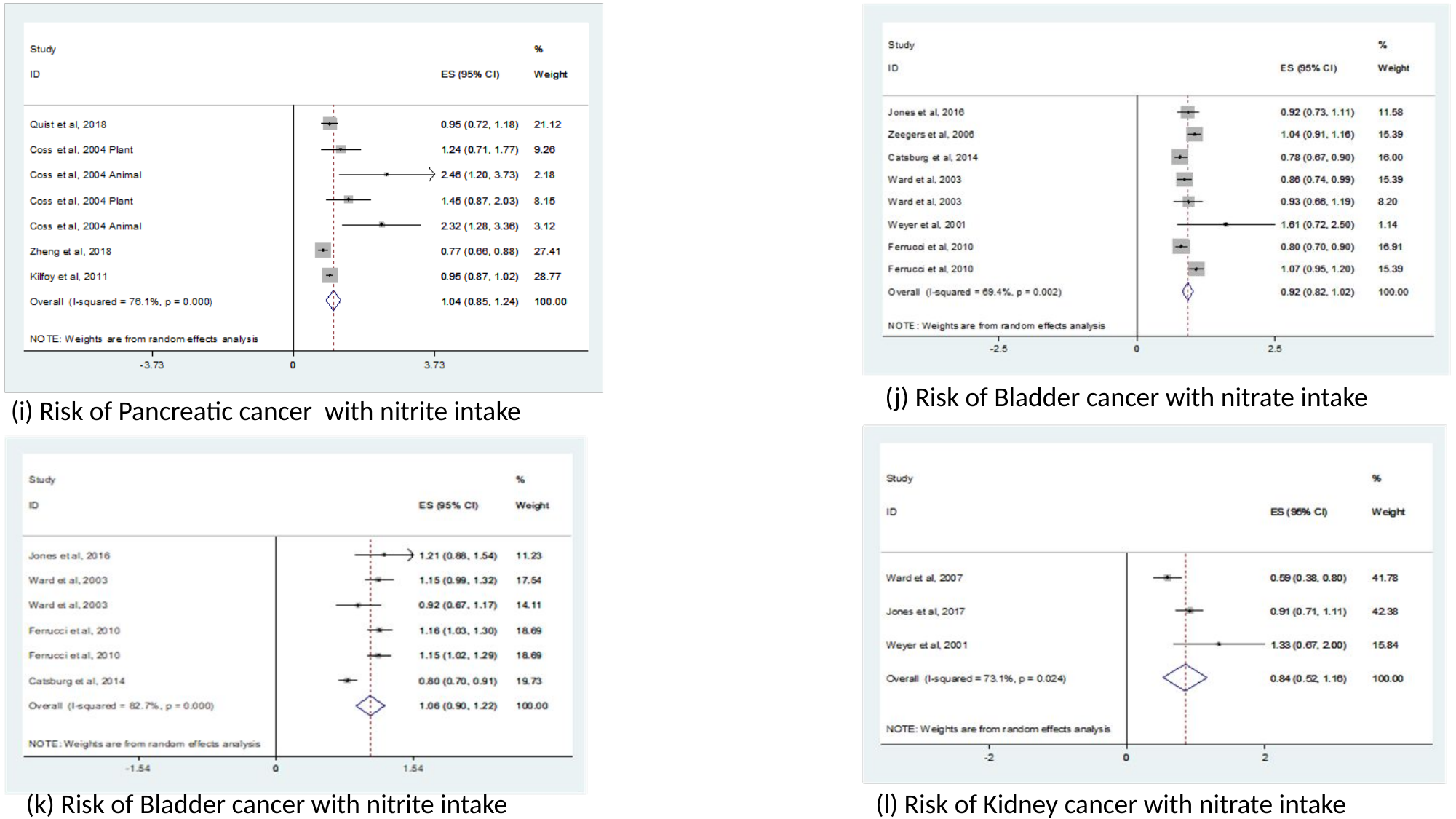

(j) Risk of Bladder cancer with nitrate intake
(i) Risk of Pancreatic cancer with nitrite intake
(k) Risk of Bladder cancer with nitrite intake
 (l) Risk of Kidney cancer with nitrate intake

## Slide 13
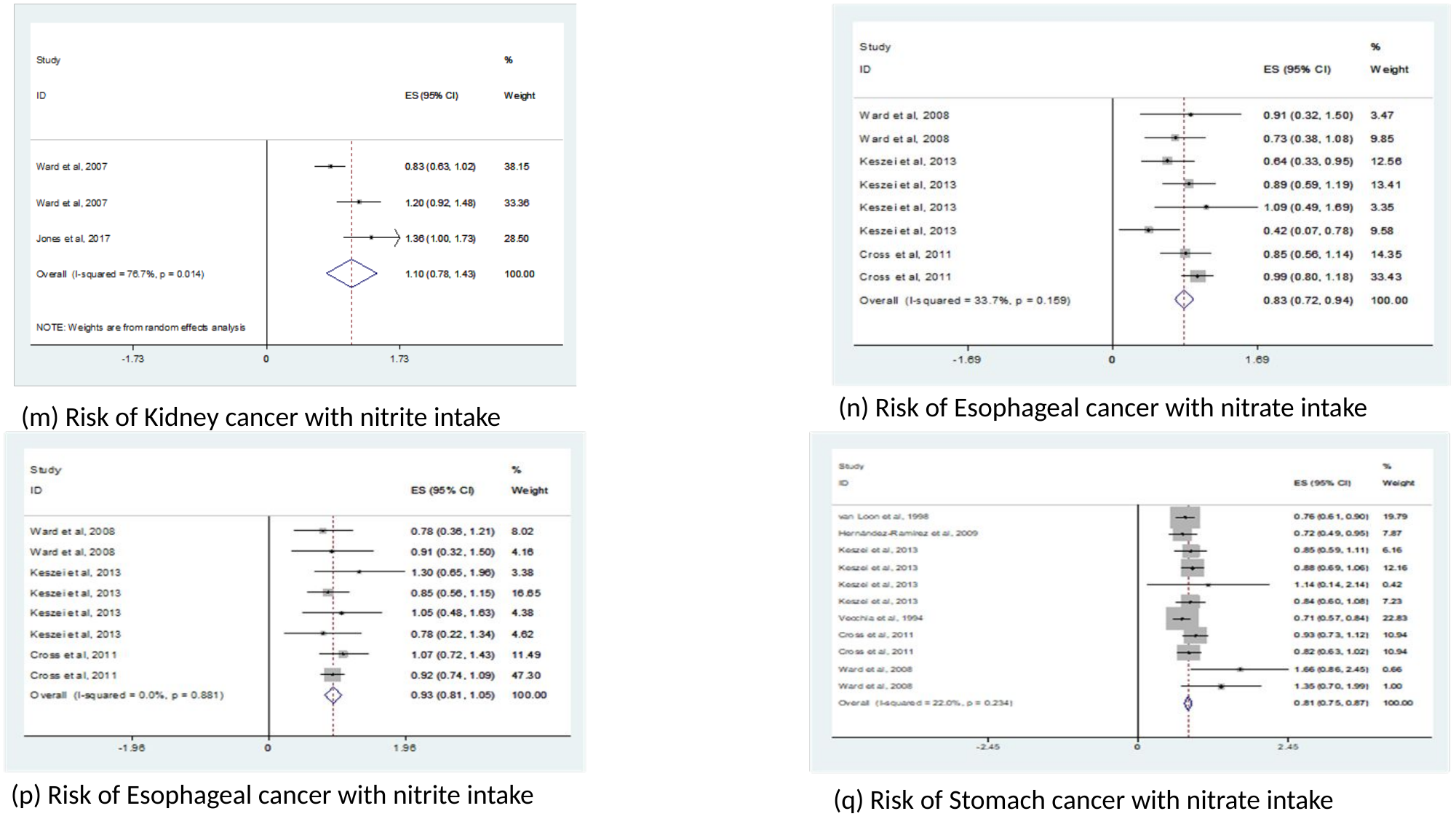

(n) Risk of Esophageal cancer with nitrate intake
(m) Risk of Kidney cancer with nitrite intake
(p) Risk of Esophageal cancer with nitrite intake
 (q) Risk of Stomach cancer with nitrate intake

## Slide 14
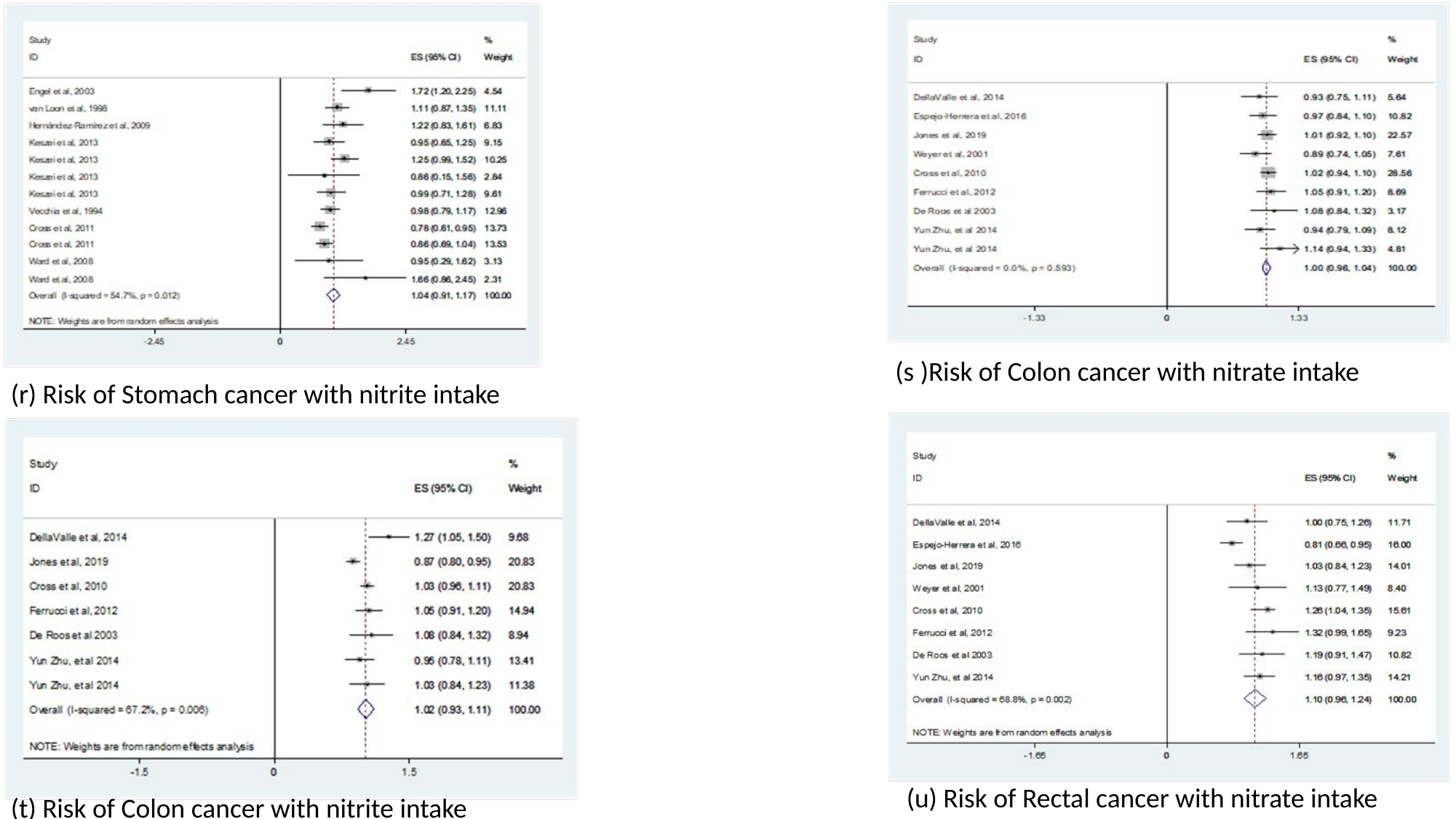

(s )Risk of Colon cancer with nitrate intake
(r) Risk of Stomach cancer with nitrite intake
 (u) Risk of Rectal cancer with nitrate intake
(t) Risk of Colon cancer with nitrite intake

## Slide 15
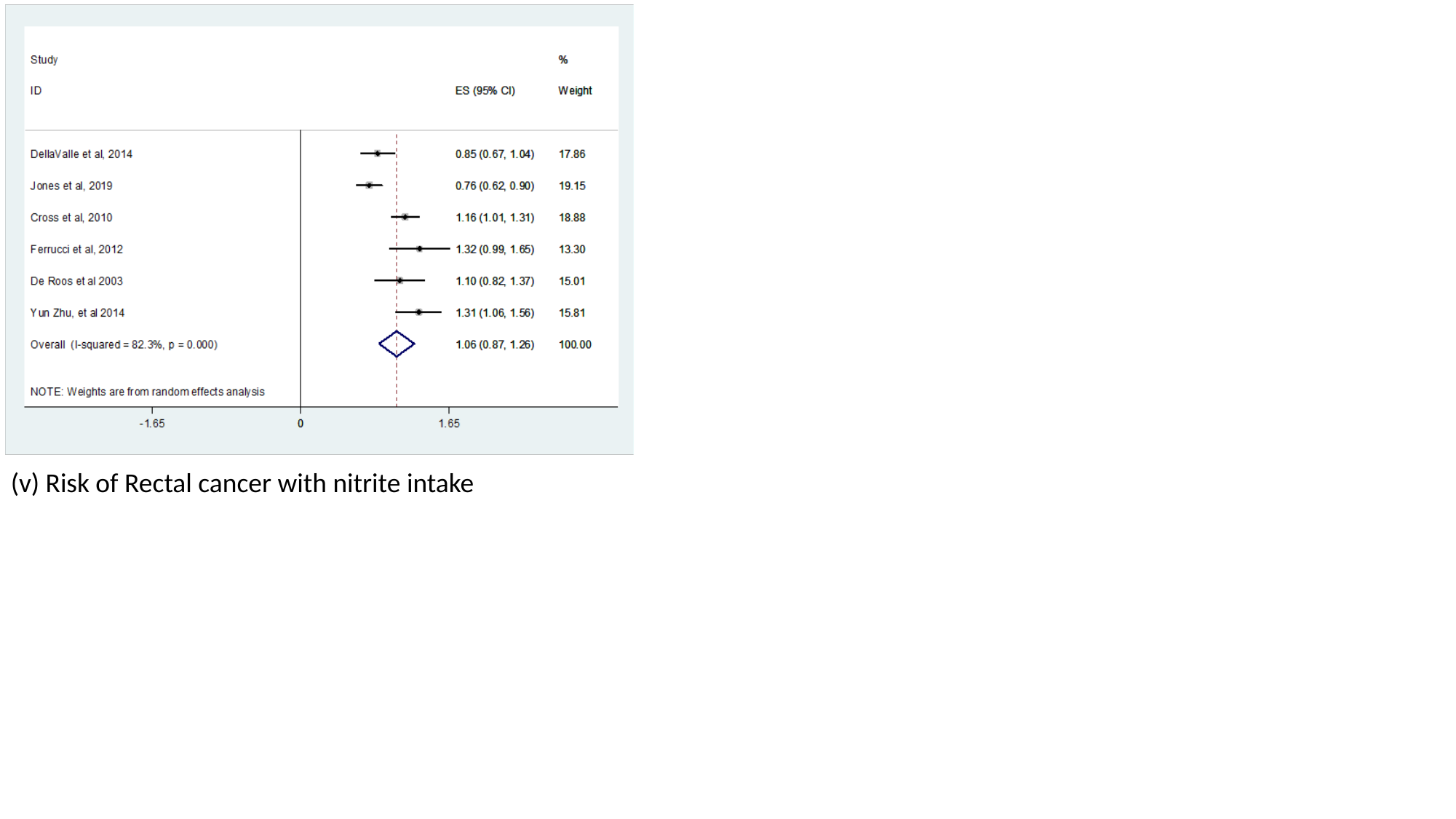

(v) Risk of Rectal cancer with nitrite intake

## Slide 16
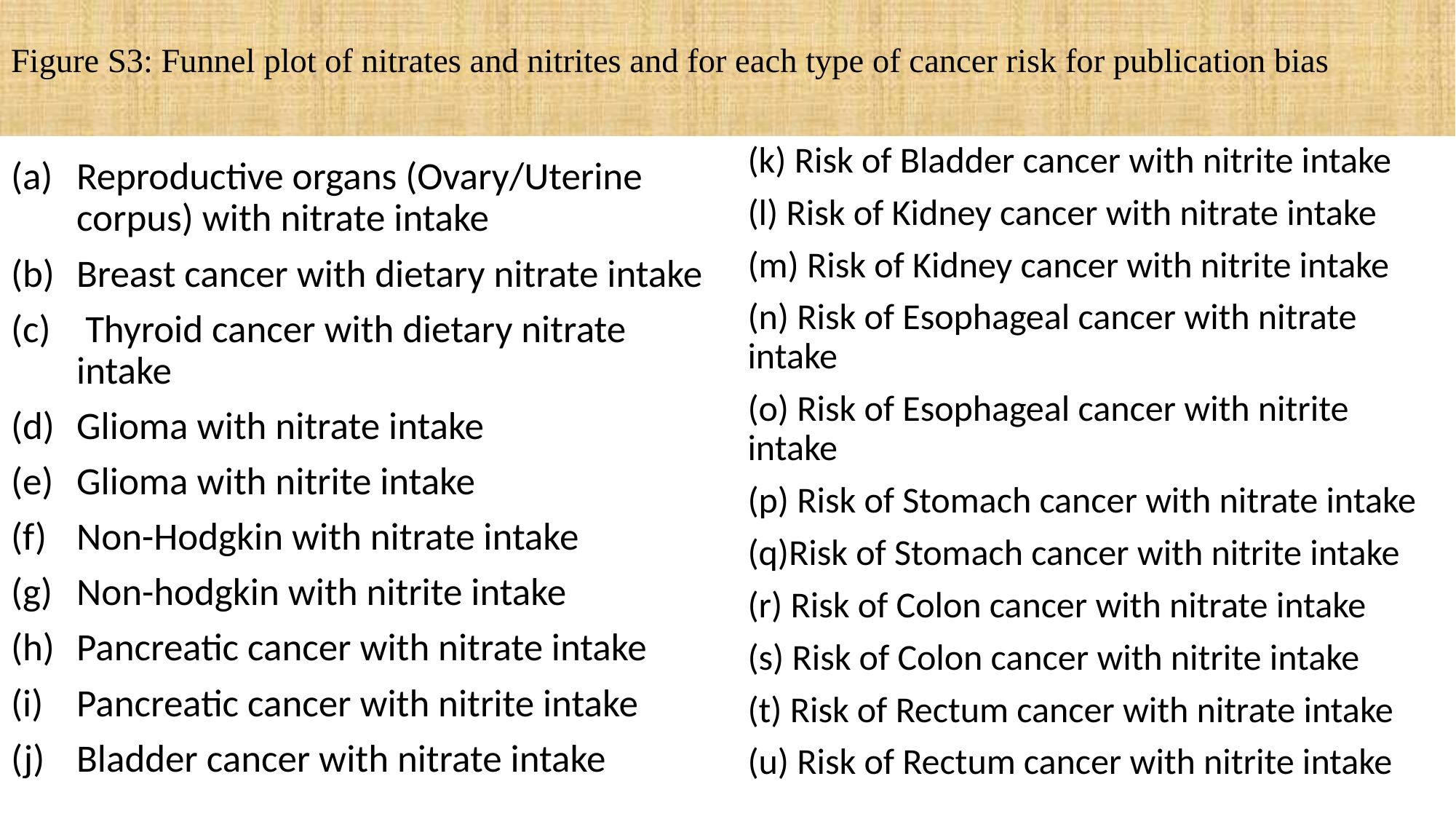

# Figure S3: Funnel plot of nitrates and nitrites and for each type of cancer risk for publication bias
(k) Risk of Bladder cancer with nitrite intake
(l) Risk of Kidney cancer with nitrate intake
(m) Risk of Kidney cancer with nitrite intake
(n) Risk of Esophageal cancer with nitrate intake
(o) Risk of Esophageal cancer with nitrite intake
(p) Risk of Stomach cancer with nitrate intake
(q)Risk of Stomach cancer with nitrite intake
(r) Risk of Colon cancer with nitrate intake
(s) Risk of Colon cancer with nitrite intake
(t) Risk of Rectum cancer with nitrate intake
(u) Risk of Rectum cancer with nitrite intake
Reproductive organs (Ovary/Uterine corpus) with nitrate intake
Breast cancer with dietary nitrate intake
 Thyroid cancer with dietary nitrate intake
Glioma with nitrate intake
Glioma with nitrite intake
Non-Hodgkin with nitrate intake
Non-hodgkin with nitrite intake
Pancreatic cancer with nitrate intake
Pancreatic cancer with nitrite intake
Bladder cancer with nitrate intake

## Slide 17
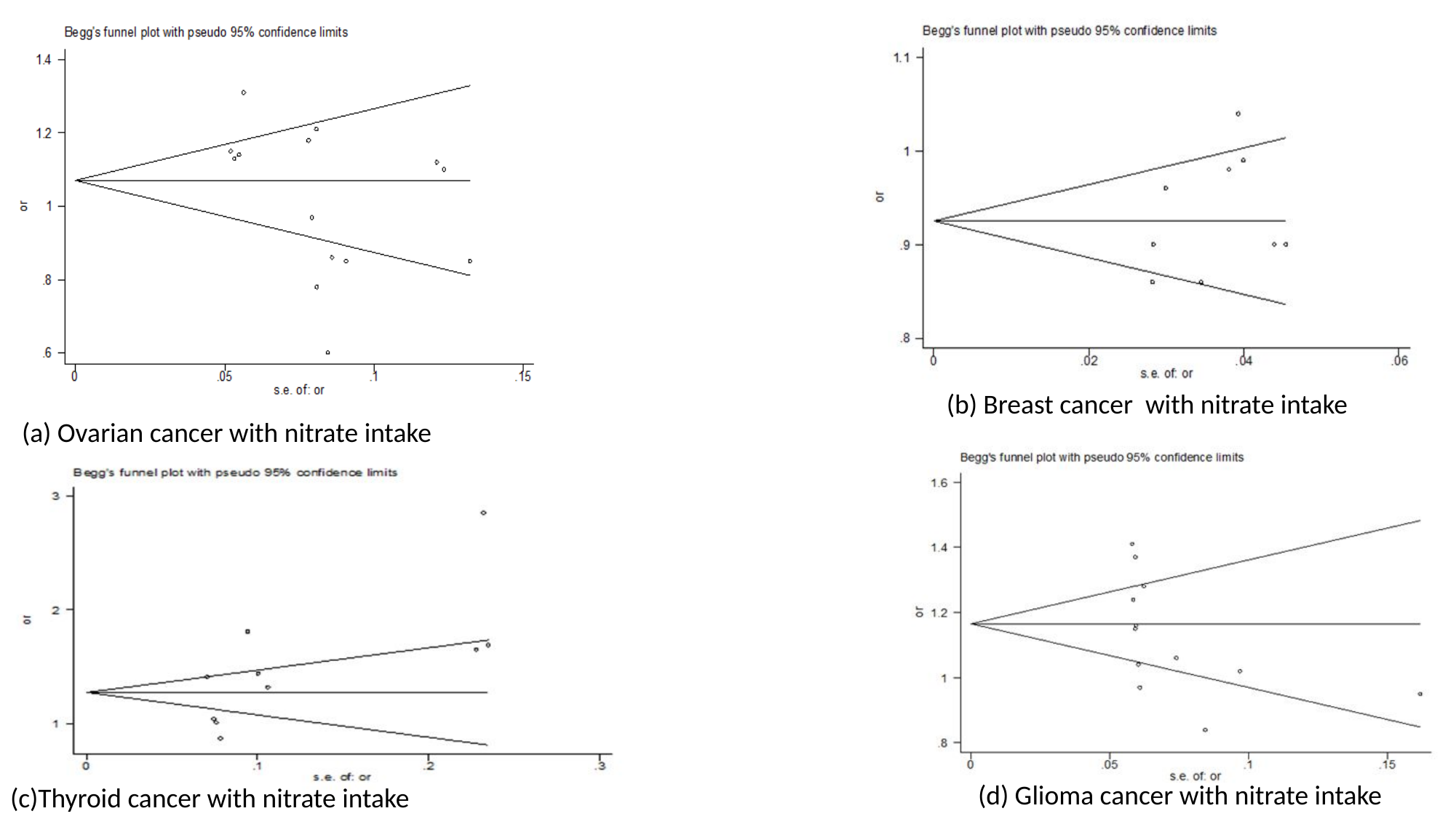

(b) Breast cancer with nitrate intake
(a) Ovarian cancer with nitrate intake
(d) Glioma cancer with nitrate intake
 (c)Thyroid cancer with nitrate intake

## Slide 18
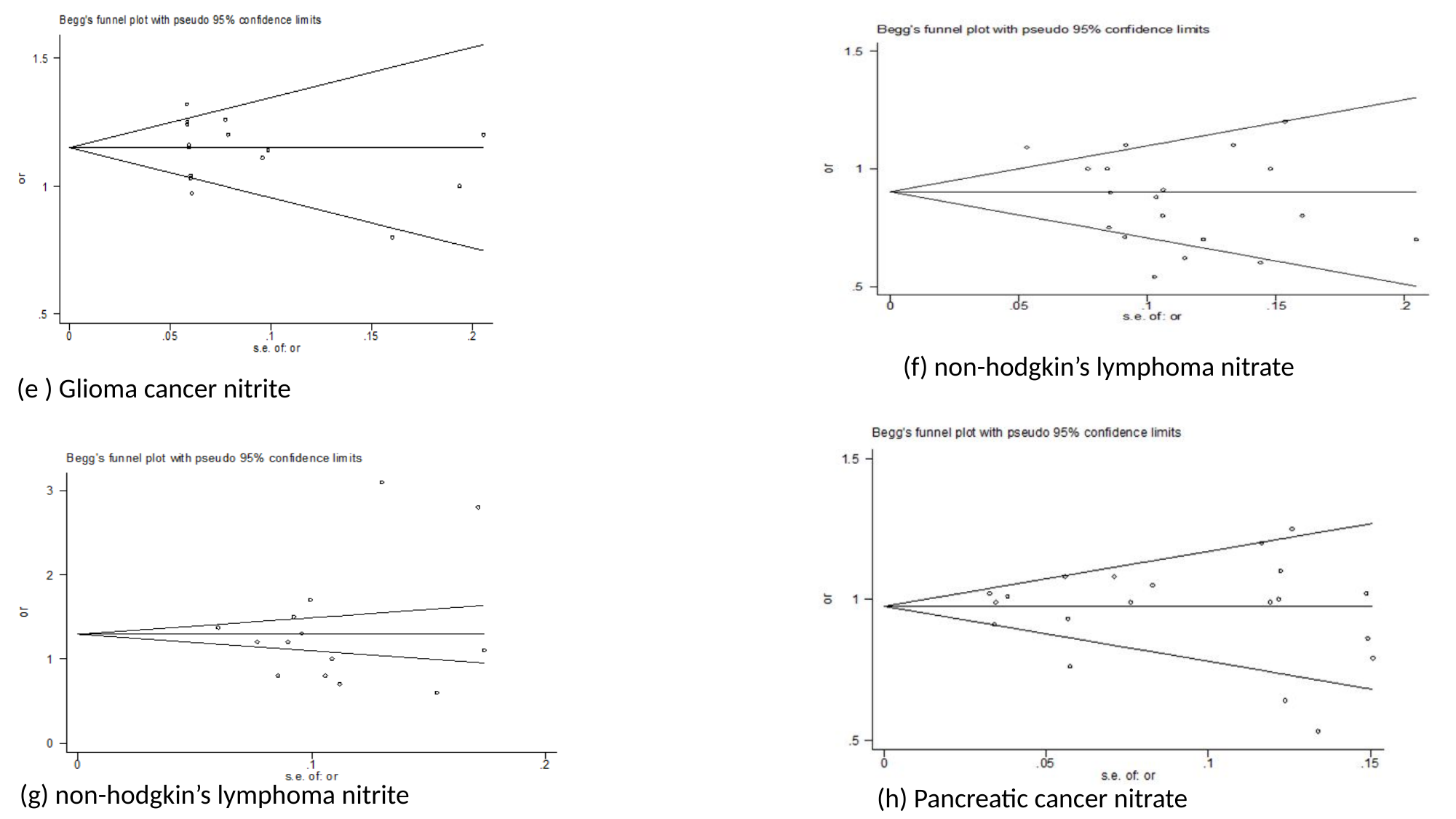

(f) non-hodgkin’s lymphoma nitrate
 (e ) Glioma cancer nitrite
(g) non-hodgkin’s lymphoma nitrite
(h) Pancreatic cancer nitrate

## Slide 19
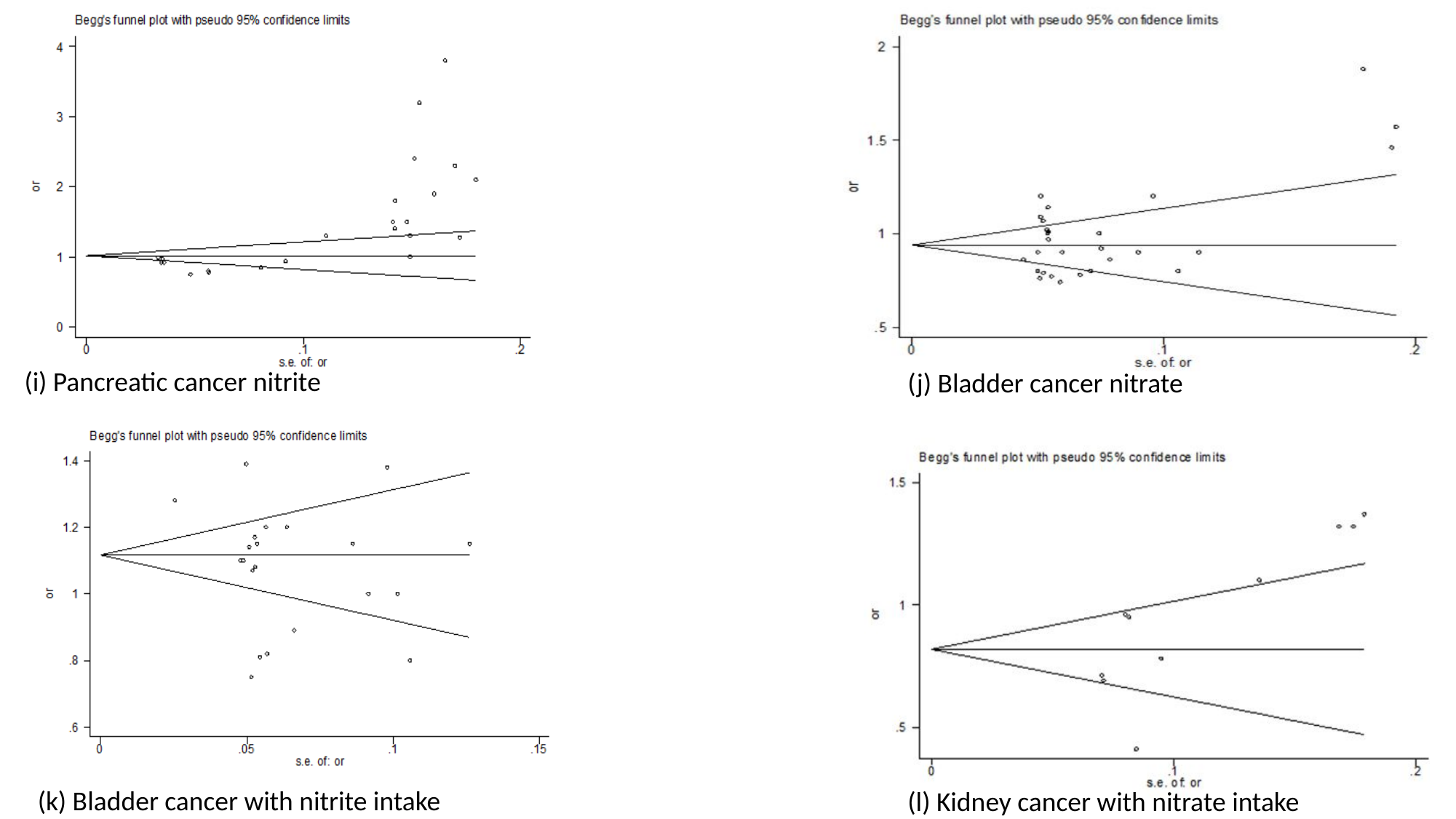

(i) Pancreatic cancer nitrite
(j) Bladder cancer nitrate
(k) Bladder cancer with nitrite intake
(l) Kidney cancer with nitrate intake

## Slide 20
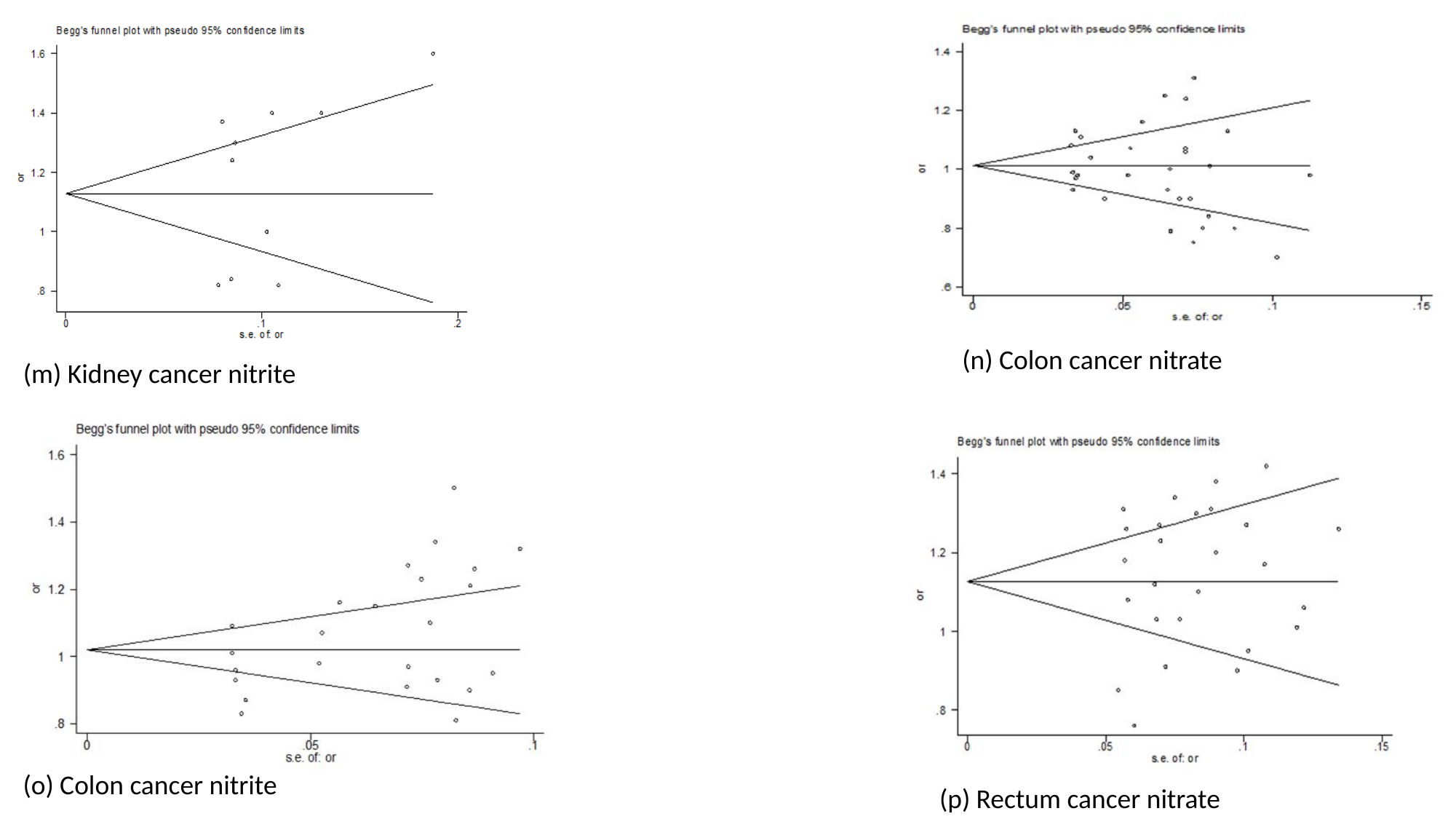

(n) Colon cancer nitrate
(m) Kidney cancer nitrite
(o) Colon cancer nitrite
(p) Rectum cancer nitrate

## Slide 21
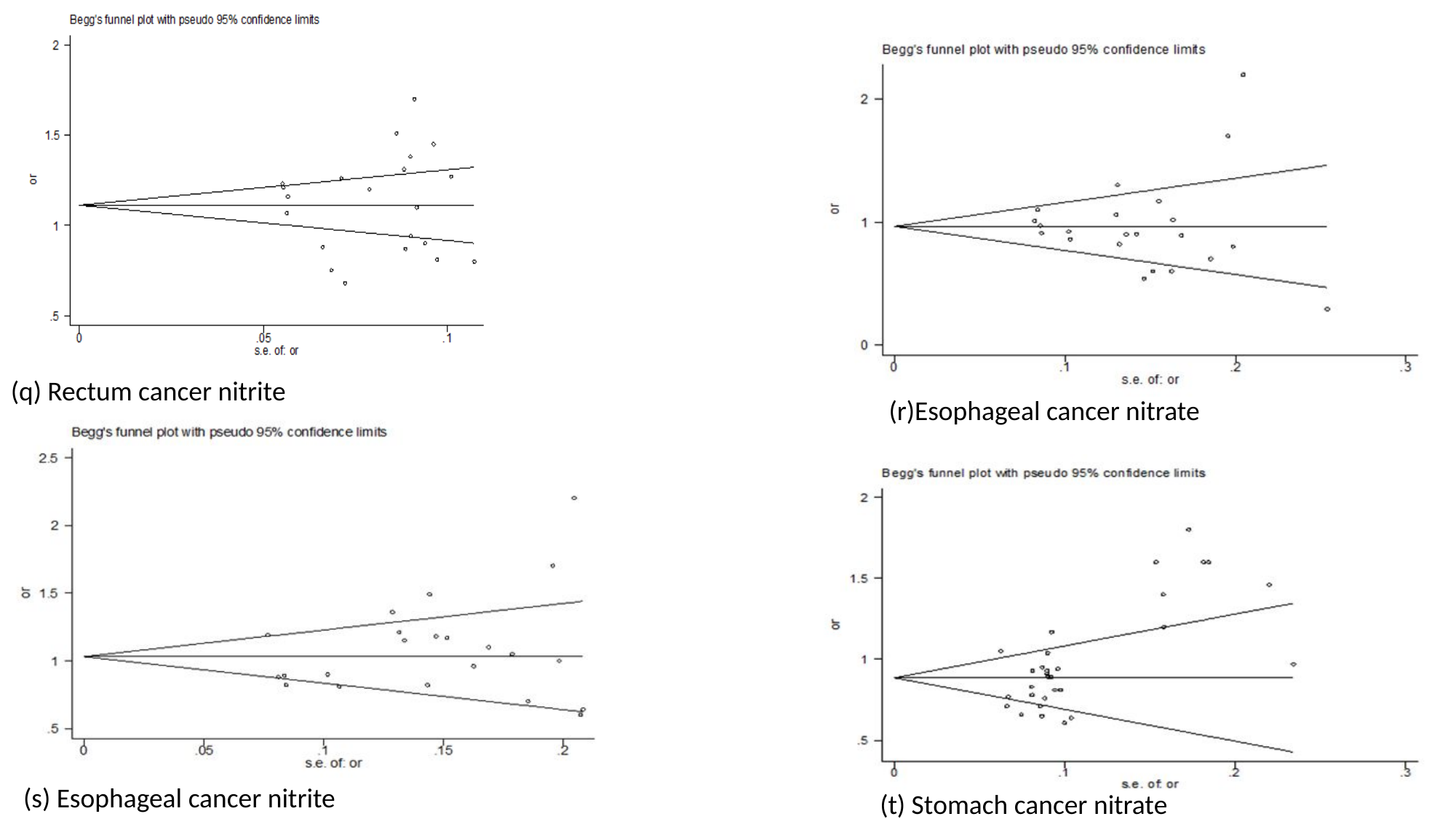

(q) Rectum cancer nitrite
(r)Esophageal cancer nitrate
(s) Esophageal cancer nitrite
 (t) Stomach cancer nitrate

## Slide 22
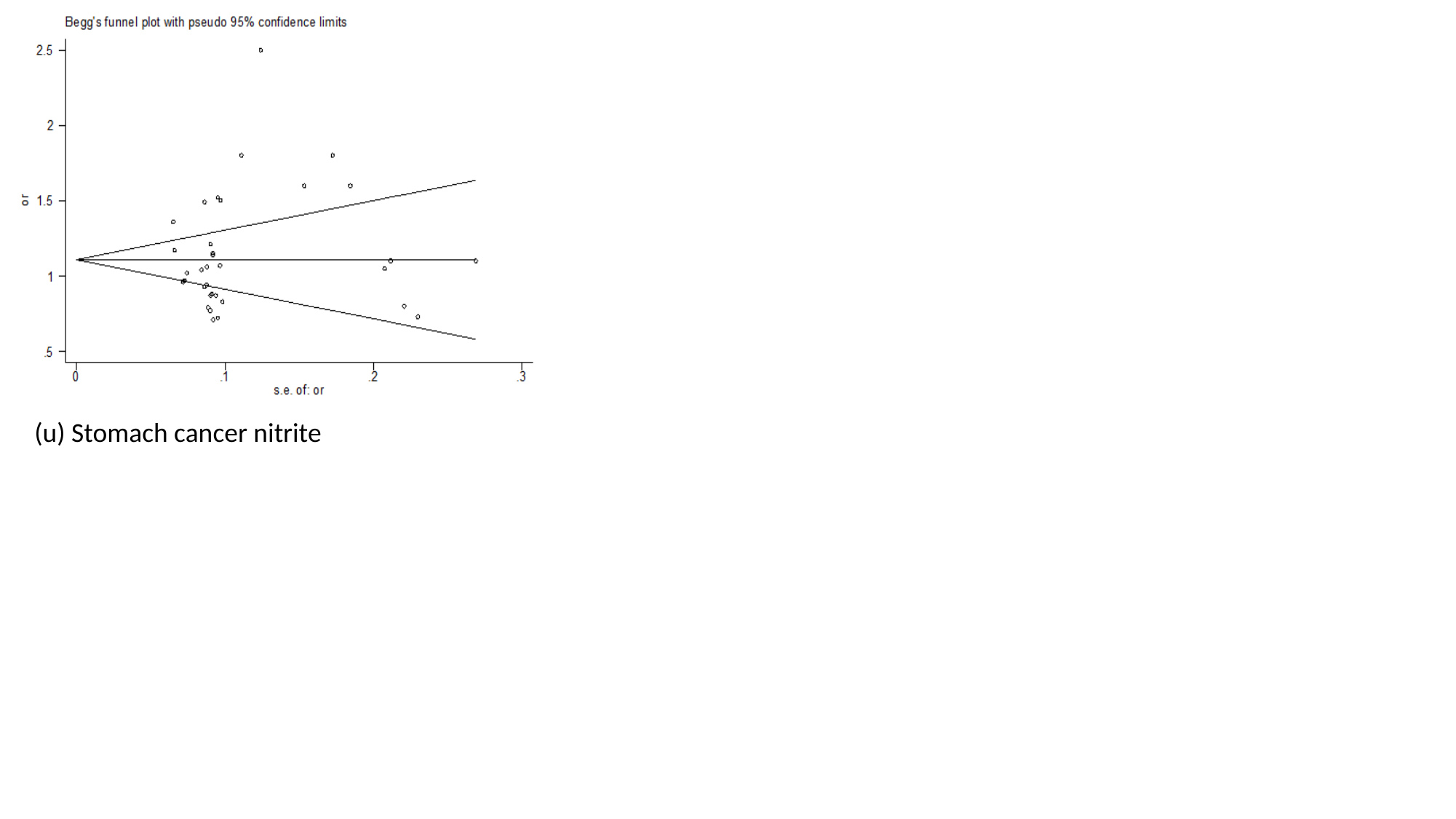

(u) Stomach cancer nitrite
